# Insulin signalling regulates *Pink1* mRNA localization via modulation of AMPK activity to support PINK1 function in neurons

**DOI:** 10.1038/s42255-024-01007-w

**Published:** 2024-03-19

**Authors:** J. Tabitha Hees, Simone Wanderoy, Jana Lindner, Marlena Helms, Hariharan Murali Mahadevan, Angelika B. Harbauer

**Affiliations:** 1 TUM Medical Graduate Center, Technical University of Munich, Munich, Germany; 2 Max Planck Institute for Biological Intelligence, Martinsried, Germany; 3 Technical University of Munich, Institute of Neuronal Cell Biology, Munich, Germany; 4 Munich Cluster for Systems Neurology, Munich, Germany

**Keywords:** Cellular neuroscience, Alzheimer's disease, Insulin signalling, Metabolism, Mitophagy

## Abstract

Mitochondrial quality control failure is frequently observed in neurodegenerative diseases. The detection of damaged mitochondria by stabilization of PTEN-induced kinase 1 (PINK1) requires transport of *Pink1* messenger RNA (mRNA) by tethering it to the mitochondrial surface. Here, we report that inhibition of AMP-activated protein kinase (AMPK) by activation of the insulin signalling cascade prevents *Pink1* mRNA binding to mitochondria. Mechanistically, AMPK phosphorylates the RNA anchor complex subunit SYNJ2BP within its PDZ domain, a phosphorylation site that is necessary for its interaction with the RNA-binding protein SYNJ2. Notably, loss of mitochondrial *Pink1* mRNA association upon insulin addition is required for PINK1 protein activation and its function as a ubiquitin kinase in the mitophagy pathway, thus placing PINK1 function under metabolic control. Induction of insulin resistance in vitro by the key genetic Alzheimer risk factor apolipoprotein E4 retains *Pink1* mRNA at the mitochondria and prevents proper PINK1 activity, especially in neurites. Our results thus identify a metabolic switch controlling *Pink1* mRNA localization and PINK1 activity via insulin and AMPK signalling in neurons and propose a mechanistic connection between insulin resistance and mitochondrial dysfunction.

## Abstract (web summary)

Hees et al. identify a mechanism that integrates insulin signalling with distal mitochondrial quality control in neurons via AMPK/PINK1, with implications for mitochondrial dysfunction in the context of insulin resistance in neurons.

## Main

Neurons are particularly challenged in maintaining and distributing mitochondria throughout neurites due to their highly extended and complex structures^1^. Transport of nuclear-encoded mRNAs for mitochondrial proteins and their local translation support the upkeep of mitochondrial functionality within neuronal processes^2^. We have recently identified a neuron-specific mechanism that tethers the transcript encoding for the mitochondrial protein PINK1 and potentially also other nuclear-encoded mitochondrial transcripts to mitochondria, thereby facilitating their transport and local translation in axons and dendrites^3^. PINK1, mutated in certain familial forms of Parkinson’s disease (PD)^4^, is a short-lived protein^5^ that functions as a sensor for mitochondrial damage. Under physiological conditions, PINK1 is imported into mitochondria, cleaved and degraded^6^. In damaged mitochondria, however, the depolarized membrane potential impairs mitochondrial PINK1 import. Instead, PINK1 stabilizes on the outer mitochondrial membrane^7^ and phosphorylates ubiquitin molecules at serine 65 leading to recruitment and partial activation of the E3 ubiquitin ligase Parkin^8^. PINK1 phosphorylates Parkin at serine 65 resulting in full activation of Parkin^9^, which then ubiquitinates several proteins on the outer mitochondrial membrane^8^. The ubiquitin molecules, in turn, are further phosphorylated by PINK1. The phosphorylated ubiquitin chains covering the damaged mitochondria are then recognized by autophagy receptors such as optineurin^10,11^. This ultimately results in the formation of autophagosomes and their delivery to degradative lysosomes^8^. The interaction between *Pink1* mRNA and mitochondria is maintained by an anchoring complex consisting of the outer mitochondrial membrane protein Synaptojanin 2 binding protein (SYNJ2BP; also called OMP25) and the phosphatidylinositol phosphatase Synaptojanin 2 (SYNJ2), which contains an RNA-binding motif^3^. SYNJ2BP is ubiquitously expressed and localized to the outer mitochondrial membrane due to its C-terminal transmembrane domain^12^. SYNJ2BP has a PDZ domain at its N terminus that specifically binds to a unique motif in the C terminus of SYNJ2a^12^, a splice variant of SYNJ2 that is primarily expressed in neurons^3^. SYNJ2a binds *Pink1* mRNA, whereas SYNJ2BP serves as mitochondrial anchor tethering SYNJ2a and *Pink1* mRNA to mitochondria, ensuring a constant supply of fresh PINK1 protein by local translation in axons and dendrites^3^. This is required to support local mitophagy in distal neurites to eliminate acutely damaged mitochondria^3^; however, it still remains to be elucidated how localization of *Pink1* mRNA as well as PINK1 activation and function are regulated in response to local signalling pathways in neurons.

AMPK is known as the master regulator of energy sensing and consequently is closely associated with mitochondrial homeostasis^13^. AMPK is composed of three subunits: one catalytic α-subunit and two regulatory β- and γ-subunits^13^. It has more than 100 known targets, including multiple proteins that are involved in various aspects of mitochondrial homeostasis such as mitochondrial biogenesis, mitochondrial fission and autophagy^13^; however, its importance in PINK1/Parkin-dependent mitophagy in neurons remains to be elucidated. AMPK is generally activated in response to an increased AMP:ATP ratio, but is also regulated by several upstream kinases, including AKT downstream of insulin receptor (IR) signalling. In response to the hormone insulin, the IR autophosphorylates and recruits IR substrate adaptor proteins^14^. Subsequently, phosphatidylinositol 3-kinase (PI3K) gets activated, thereby inducing AKT activity. AKT in turn inhibits AMPK by phosphorylating the catalytic α-subunit^15^. Insulin signalling has also been shown to regulate neuronal mitochondrial function, including ATP production, respiration and calcium buffering as well as protein biogenesis and homeostasis^16^. Of note, insulin resistance in the brain is associated with mitochondrial dysfunction^16^ and fittingly, type 2 diabetes represents a risk factor for neurodegenerative diseases, including PD and Alzheimer’s disease (AD)^17^. The strongest genetic risk factor for AD is the presence of the ε4 allele of apolipoprotein E (ApoE)^18^, which has mechanistically been linked to decreased insulin signalling and insulin resistance in the brain by trapping the IR in early endosomes^19^, yet how ApoE4 presence affects mitophagy remains to be determined.

In this study, we aimed to investigate how insulin and AMPK signalling regulate mitochondrial *Pink1* mRNA localization and PINK1 function. We identified SYNJ2BP as a substrate of AMPK downstream of insulin signalling. While SYNJ2BP phosphorylation is required for *Pink1* mRNA localization to mitochondria, insulin-induced AMPK inhibition and subsequent dissociation of *Pink1* mRNA from mitochondria promotes PINK1 protein activation. Disruption of insulin signalling by ApoE4 leads to impaired PINK1 activation in cultured neurons and therefore suggests a mechanistic connection between the frequently observed neurodegenerative phenotypes of insulin resistance and mitochondrial dysfunction.

## Results

### AMPK regulates *Pink1* mRNA localization to mitochondria

**Fig. 1:**
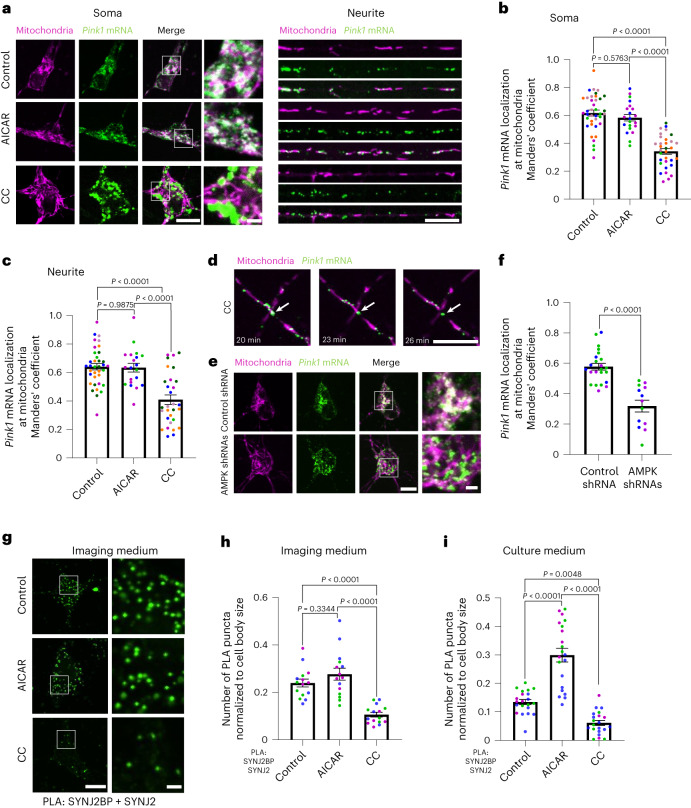
AMPK signalling regulates *Pink1* mRNA localization to mitochondria. **a**, Representative images of *Pink1* mRNA visualized by the MS2/PP7-split-Venus method and mitoRaspberry upon AMPK activation and inhibition using AICAR (1 mM, 2 h) and CC (20 µM, 2 h), respectively, in the soma and neurites. **b**, Quantification of the Manders’ colocalization coefficient for the overlap between the *Pink1* mRNA and mitochondrial channel in the soma as in **a**. One-way ANOVA followed by Tukey’s post hoc test; *n* = 21–38. **c**, Quantification as in **b** for neurites. One-way ANOVA followed by Tukey’s post hoc test; *n* = 20–36. **d**, Representative images of *Pink1* mRNA visualized by the MS2/PP7-split-Venus method and mitoRaspberry 20 min, 23 min and 26 min after 20 µM CC treatment. **e**, Representative images of *Pink1* mRNA and mitoRaspberry upon control or AMPK shRNA expression in the soma. **f**, Quantification of the Manders’ colocalization coefficient for the overlap between the *Pink1* mRNA and mitochondrial channel as in **e**. Two-tailed Student’s *t-*test; *n* = 13–23. **g**, Representative images of neuronal somata displaying the PLA signal between SYNJ2BP and SYNJ2 upon control, AICAR (1 mM, 2 h) and CC (20 µM, 2 h) treatment in imaging medium (Hibernate E). **h**, Number of PLA puncta per soma of neurons normalized to the soma size treated with the indicated drugs as in **g** in imaging medium (Hibernate E). One-way ANOVA followed by Tukey’s post hoc test; *n* = 15. **i** Quantification as in **h** of neurons grown in full culture medium and treated with the indicated drugs. One-way ANOVA followed by Tukey’s post hoc test; *n* = 21. All data are expressed as mean ± s.e.m. All data points represent single cells coming from at least three biological replicates. Scale bars, 10 µm; scale bars in insets, 2 µm. Source data

**Extended Data Fig. 1:**
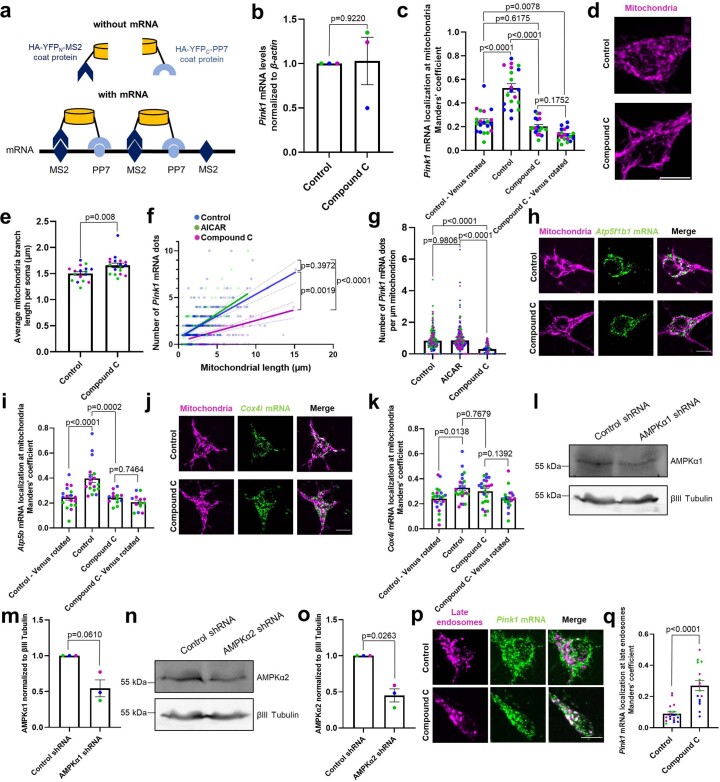
AMPK signalling regulates *Pink1* mRNA localization to mitochondria. **a** Schematic of the MS2/PP7-split-Venus method for mRNA imaging. **b** RT-qPCR of *Pink1* transcript levels normalized to β-*actin* from primary cortical neurons treated with or without CC (20 µM, 2 h). Two-tailed Welch’s t-test; n = 3. **c** Live-cell imaging of *Pink1* mRNA localization in hippocampal neurons using the MS2/PP7-split-Venus method. Quantification of the Manders’ colocalization coefficient for the overlap between the *Pink1* mRNA and the mitochondrial channel upon control or CC (20 µM, 2 h) treatment. The analysis has been performed on a 10 by 10 µm square in the soma. ‘Venus rotated’ indicates that the *Pink1* mRNA channel had been rotated 90° before quantification. One-way ANOVA followed by Tukey’s post hoc test; n = 19-20. **d** Representative images of neurons overexpressing mito-meGFP upon control or CC (20 µM, 2 h) treatment. **e** Quantification of the average mitochondrial branch length per soma as in d. Two-tailed student’s t-test; n = 18. **f** Number of *Pink1* mRNA dots plotted against the mitochondrial length in µm upon control, AICAR (1 mM, 2 h) or CC (20 µM, 2 h) treatment. Simple linear regression; one-way ANOVA followed by Tukey’s post hoc test to compare the slopes of all three groups; n = 140-218. **g** Number of *Pink1* mRNA dots per µm mitochondrion upon control, AICAR (1 mM, 2 h) or CC (20 µM, 2 h) treatment. One-way ANOVA followed by Tukey’s post hoc test; n = 167-310. **h** Representative images of *Atp5f1b* mRNA visualized by the MS2/PP7-split-Venus method and mitoRaspberry upon control or CC (20 µM, 2 h) treatment. **i** Quantification of the Manders’ colocalization coefficient for the overlap between the *Atp5f1b* mRNA and the mitochondrial channel as in h. The analysis has been performed on a 10 by 10 µm square in the soma. ‘Venus rotated’ indicates that the *Atp5f1b* mRNA channel had been rotated 90° before quantification. One-way ANOVA followed by Tukey’s post hoc test; n = 13-18. **j** Representative images of *Cox4i* mRNA visualized by the MS2/PP7-split-Venus method and mitoRaspberry upon control or CC (20 µM, 2 h) treatment. **k** Quantification of the Manders’ colocalization coefficient for the overlap between the *Cox4i* mRNA and the mitochondrial channel as in j. The analysis has been performed on a 10 by 10 µm square in the soma. ‘Venus rotated’ indicates that the *Cox4i* mRNA channel had been rotated 90° before quantification. One-way ANOVA followed by Tukey’s post hoc test; n = 23. **l** Representative immunoblot images of cortical neurons lentivirally overexpressing control shRNA or AMPKα1 shRNA. **m** Quantification of the AMPKα1 protein bands normalized to the respective βIII tubulin bands as in l. Two-tailed Welch’s t-test; n = 3. **n** Representative immunoblot images of cortical neurons lentivirally overexpressing control shRNA or AMPKα2 shRNA. **o** Quantification of the AMPKα2 protein bands normalized to the respective βIII tubulin bands as in m. Two-tailed Welch’s t-test; n = 3. **p** Representative images of *Pink1* mRNA visualized by the MS2/PP7-split-Venus method and iRFP-Rab7 upon control or CC (20 µM, 2 h) treatment. **q** Quantification of the Manders’ colocalization coefficient for the overlap between the *Pink1* mRNA and iRFP-Rab7 channel as in p. Two-tailed student’s t-test; n = 16-19. All data are expressed as mean ± SEM. Data points represent biological replicates (**b, m, o**), single cells (**c, e, i, k, q**) or single mitochondria (**f, g**) coming from ≥3 biological replicates. Scale bars, 10 µm. Source data

To test whether mitochondrial localization of the *Pink1* mRNA is influenced by cellular metabolism, we treated mouse hippocampal neurons grown in vitro with either the AMP analogue 5-aminoimidazole-4-carboxamide ribonucleoside (AICAR) or Compound C (CC), which have been shown to activate and inhibit the master regulator kinase AMPK, respectively. To evaluate the impact of AICAR and CC on *Pink1* mRNA localization, we visualized mitochondria by expressing mitochondrially targeted mRaspberry and *Pink1* mRNA using the MS2/PP7-split-Venus system that we established previously^3^. In brief, this technique employs the high affinity interaction between two phage-derived capsid proteins (MS2 and PP7 coat proteins) and their respective RNA stem loops (MS2 and PP7), which we inserted downstream of the rat Pink1 3′ untranslated region (UTR) in 12 alternating copies. Coexpression of the so-tagged *Pink1* mRNA together with the coat proteins, each fused to one half of split Venus, allows for the specific and background-free labelling of RNA in living cells^20^ (Extended Data Fig. 1a). While AICAR treatment had no effect on mitochondrial *Pink1* mRNA association, CC led to a loss of colocalization of *Pink1* mRNA with mitochondria in both the soma and neurites (Fig. 1a), which we quantified using Manders’ coefficient (Fig. 1b,c). Time-lapse imaging showed that *Pink1* mRNA dissociates from mitochondria 20 min after CC treatment (Fig. 1d and Supplementary Videos 1–3). Furthermore, we confirmed by qPCR that the loss of colocalization was not due to a reduction of *Pink1* mRNA levels upon CC treatment (Extended Data Fig. 1b). We also determined the colocalization in a square within the cell body before and after 90° rotation of one imaging channel. The association between *Pink1* mRNA and mitochondria under control conditions was significantly higher than its corresponding rotated quantification, whereas the association in the presence of CC was similar to chance levels, represented by its corresponding rotated quantification (Extended Data Fig. 1c). This indicates a CC-specific effect on mitochondrial *Pink1* mRNA localization. AMPK has previously been shown to affect mitochondrial morphology^21^. Accordingly, we observed an increase in mitochondrial branch length in neuronal somata upon CC treatment (Extended Data Fig. 1d,e). To exclude the possibility that this altered morphology affected our quantification, we determined the length of mitochondria in neurites and the number of *Pink1* mRNA dots per mitochondrion. As expected, longer mitochondria had more *Pink1* mRNA dots (Extended Data Fig. 1f). The amount of *Pink1* mRNA dots per µm mitochondrion decreased irrespective of mitochondrial length after CC treatment compared to control and AICAR treatment (Extended Data Fig. 1f,g) arguing that this observation is independent of the effect of AMPK on mitochondrial length. To test whether this is a response specific to SYNJ2a and SYNJ2BP substrates, we tested two other transcripts that we had previously shown to colocalize with neuronal mitochondria^3^, *Atp5f1b* and *Cox4i* mRNA, of which only *Atp5f1b* is a SYNJ2a interactor^3^. Consistently, upon CC treatment the mitochondrial association of the *Atp5f1b* transcript decreased, whereas *Cox4i* remained unaffected (Extended Data Fig. 1h–k) indicating that the regulation is specific to SYNJ2a targets. As CC is known to also affect other kinases^22^, we knocked down both isoforms of the catalytic subunit α of AMPK using short hairpin RNAs (shRNAs; as described previously^23^; Extended Data Fig. 1l–o). Loss of AMPK activity by shRNA also reduced the mitochondrial localization of *Pink1* mRNA as measured in the soma (Fig. 1e,f), fully recapitulating the effect of CC. As late endosomes have been shown to serve as translation platforms for nuclear-encoded mitochondrial transcripts in neurons^24^, we investigated whether *Pink1* mRNA localization shifted from mitochondria to Rab7-positive late endosomes upon CC treatment. As we reported earlier, late endosomal *Pink1* mRNA association is very low under control conditions^3^; however, it significantly increased after CC treatment (Extended Data Fig. 1p,q). This suggests a translocation of *Pink1* mRNA from mitochondria to late endosomes upon AMPK inhibition. Taken together, we identified AMPK as a positive regulator of mitochondrial *Pink1* mRNA localization.

**Extended Data Fig. 2:**
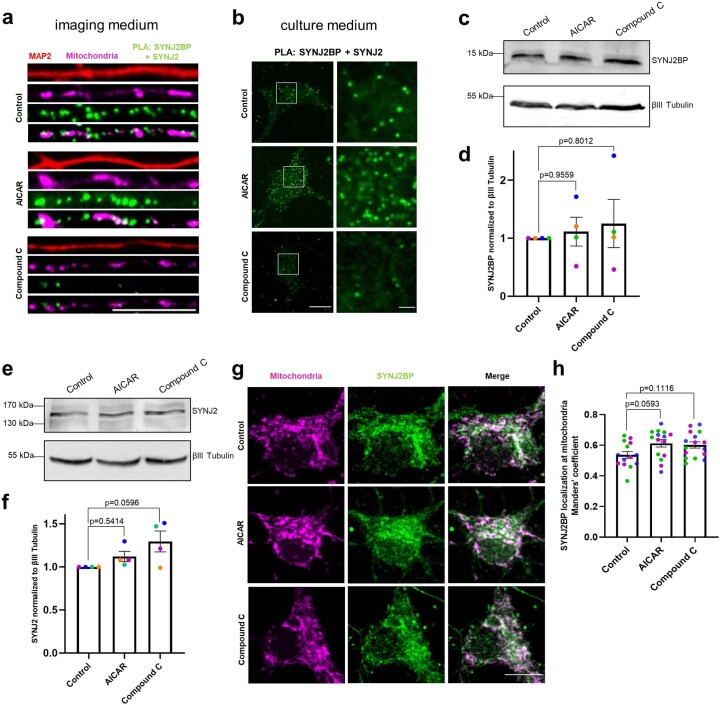
AMPK signalling regulates interaction between SYNJ2BP and SYNJ2 without affecting their expression levels. **a** Representative images of MAP2-positive dendrites displaying the PLA and mitotracker signal upon control, AICAR (1 mM, 2 h) or CC (20 µM, 2 h) treatment in imaging medium (Hibernate E). **b** Representative images of neuronal somata displaying the PLA signal upon control, AICAR (1 mM, 2 h) or CC (20 µM, 2 h) treatment in full culture medium. **c** Representative immunoblot images of cortical neurons upon control, AICAR (1 mM, 2 h) or CC (20 µM, 2 h) treatment. **d** Quantification of the SYNJ2BP protein bands normalized to the respective βIII tubulin bands as in c. One-way ANOVA followed by Tukey’s post hoc test; n = 4. **e** Representative immunoblot images of cortical neurons upon control, AICAR (1 mM, 2 h) or CC (20 µM, 2 h) treatment. **f** Quantification of the SYNJ2 protein bands normalized to the respective βIII tubulin bands as in e. One-way ANOVA followed by Tukey’s post hoc test; n = 4. **g** Representative images of neurons overexpressing mito-meGFP upon control, AICAR (1 mM, 2 h) or CC (20 µM, 2 h) treatment and stained with an antibody against SYNJ2BP. **h** Quantification of SYNJ2BP localization to mitochondria using the Manders’ colocalization coefficient as in g. One-way ANOVA followed by Tukey’s post hoc test; n = 14-16. All data are expressed as mean ± SEM. All data points represent biological replicates (**d,f**) or single cells coming from ≥3 biological replicates (**h**). Scale bars, 10 µm. Source data

To better understand the underlying mechanism, we investigated the proximity of the mitochondrial anchor SYNJ2BP to the RNA-binding protein SYNJ2 by proximity ligation assay (PLA) using antibodies to detect the endogenous proteins^3^. In line with a loss of *Pink1* mRNA tethering to mitochondria, we observed a loss of proximity between SYNJ2 and SYNJ2BP upon CC treatment (Fig. 1g and Extended Data Fig. 2a), quantified by the somatic count of PLA dots normalized to the cell body size (Fig. 1h). Notably, when we performed the experiment under similar conditions as our live-cell imaging by using Hibernate E medium instead of B27-supplemented Neurobasal medium, we were not able to further increase the number of PLA dots by activation of AMPK using AICAR (Fig. 1g,h and Extended Data Fig. 2a), which is in line with the lack of effect of AICAR treatment on *Pink1* mRNA localization (Fig. 1a–c); however, when the experiment was performed in B27 containing culture medium instead of switching to Hibernate E imaging medium, the baseline number of PLA dots dropped and was modulated in opposite directions by treatment with either AICAR or CC (Fig. 1i and Extended Data Fig. 2b). AICAR and CC treatment had no effect on the expression levels of endogenous SYNJ2BP and SYNJ2 (Extended Data Fig. 2c–f) nor the mitochondrial localization of SYNJ2BP (Extended Data Fig. 2g,h). Together, this suggests that the AMPK-dependent mechanism tethering *Pink1* mRNA to mitochondria responds to cues differentially present in Hibernate E medium and B27-supplemented Neurobasal medium.

### Insulin regulates *Pink1* mRNA localization upstream of AMPK

**Fig. 2:**
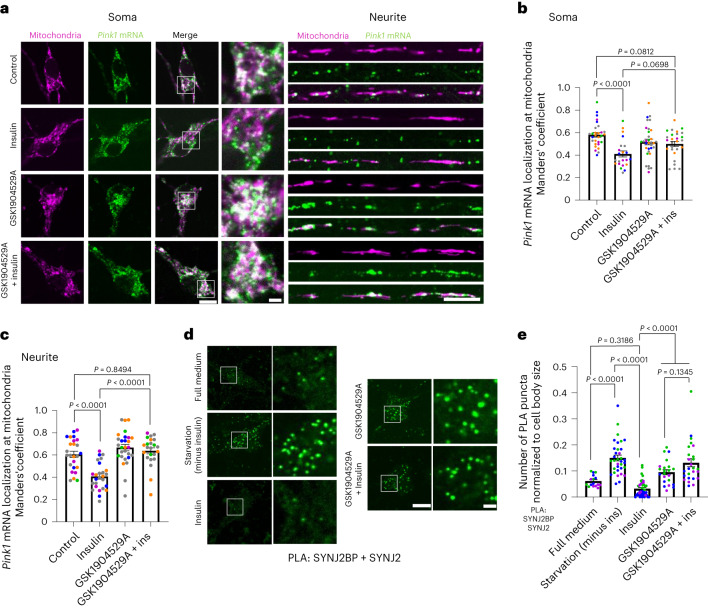
Insulin signalling regulates *Pink1* mRNA localization to mitochondria. **a**, Representative images of *Pink1* mRNA visualized by the MS2/PP7-split-Venus method and mitoRaspberry upon insulin (500 nM, 1 h) addition with or without pre-treatment with the IR inhibitor GSK1904529A (1 µM, 2 h) in the soma and neurites. **b**, Quantification of the Manders’ colocalization coefficient for the overlap between the *Pink1* mRNA and mitochondrial channel in the soma. One-way ANOVA followed by Tukey’s post hoc test; *n* = 22–29. **c**, Quantification as in **b** for neurites. One-way ANOVA followed by Tukey’s post hoc test; *n* = 23–28. **d**, Representative images of neuronal somata analysed by PLA between SYNJ2BP and SYNJ2 in full medium upon insulin starvation and upon additional insulin (500 nM, 1 h) treatment with or without pre-treatment with the IR inhibitor GSK1904529A (1 µM, 2 h). **e**, Number of PLA puncta per soma of neurons normalized to the soma size treated with the indicated drugs as in **d** in full medium or upon insulin withdrawal. One-way ANOVA followed by Tukey’s post hoc test; *n* = 16–45. All data are expressed as mean ± s.e.m. All data points represent single cells coming from at least three biological replicates. Scale bars, 10 µm; scale bars in insets; 2 µm. Source data

**Extended Data Fig. 3:**
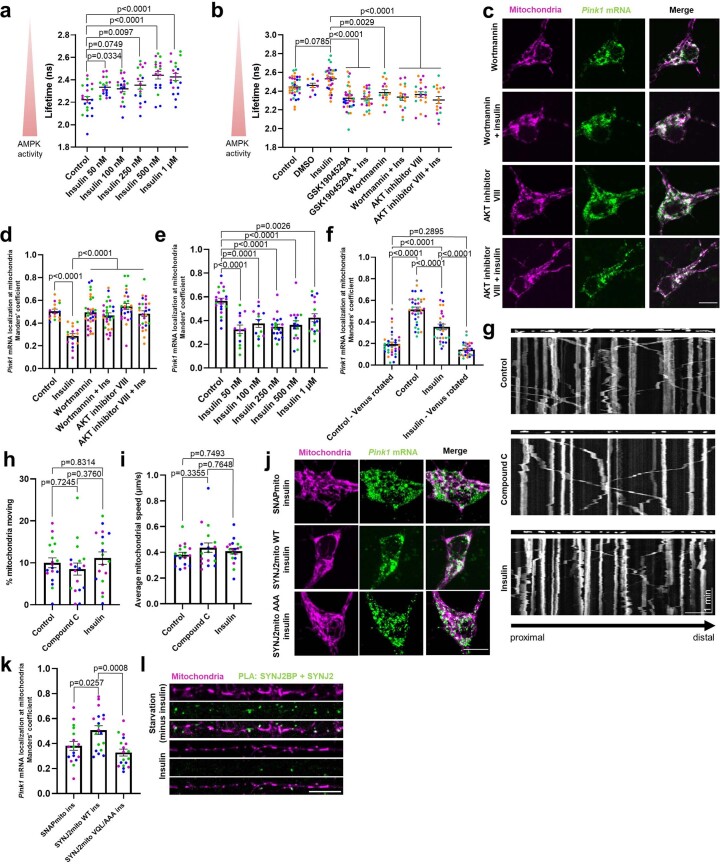
Insulin signalling inhibits AMPK and regulates *Pink1* mRNA localization to mitochondria. **a** Quantification of fluorescent lifetime imaging of the FRET-based AMPK activity sensor in neurons treated with increasing insulin concentrations for 1 h. One-way ANOVA followed by Dunnett’s post hoc test; n = 18-21. **b** Quantification of fluorescent lifetime imaging of the FRET-based AMPK activity sensor in neurons treated with insulin in presence or absence of inhibitors of the insulin signalling pathway: IR inhibitor GSK1904529A (1 µM, 2 h), PI3K inhibitor Wortmannin (1 µM, 2 h) and AKT inhibitor VIII (10 µM, 2 h). One-way ANOVA followed by Tukey’s post hoc test; n = 10-34. **c** Representative images of *Pink1* mRNA visualized by the MS2/PP7-split Venus method and mitoRaspberry upon insulin (500 nM, 1 h) addition with or without pre-treatment with the PI3K inhibitor Wortmannin (1 µM, 2 h) or the AKT inhibitor VIII (10 µM, 2 h) in the soma. **d** Quantification of the Manders’ colocalization coefficient for the overlap between the *Pink1* mRNA and mitochondrial channel in the soma. One-way ANOVA followed by Tukey’s post hoc test; n = 16-32. **e** Quantification of the Manders’ colocalization coefficient for the overlap between the *Pink1* mRNA and mitochondrial channel in neurons treated with increasing insulin concentrations for 1 h. One-way ANOVA followed by Dunnett’s post hoc test; n = 13-21. **f** Live-cell imaging of *Pink1* mRNA localization in hippocampal neurons using the MS2/PP7-split Venus method. Quantification of the Manders’ colocalization coefficient for the overlap between the *Pink1* mRNA and the mitochondrial channel upon insulin (500 nM, 1 h) treatment. The analysis has been performed on a 10 by 10 µm square in the soma. ‘Venus rotated’ indicates that the *Pink1* mRNA channel had been rotated 90° before quantification. One-way ANOVA followed by Tukey’s post hoc test; n = 30-35. **g** Representative kymographs of mitochondria in neurites of neurons overexpressing mito-meGFP upon control, CC (20 µM, 2 h) or insulin (500 nM, 1 h) treatment. **h** Quantification of the percentage of moving mitochondria in neurites as in g. One-way ANOVA followed by Tukey’s post hoc test; n = 18. **i** Quantification of the average mitochondrial speed in neurites as in g. One-way ANOVA followed by Tukey’s post hoc test; n = 18. **j** Representative images of *Pink1* mRNA visualized by the MS2/PP7-split Venus method and mitoRaspberry in neurons overexpressing SNAPmito, SYNJ2mito WT or SYNJ2mito VQL/AAA upon insulin (500 nM, 1 h) treatment. **k** Quantification of the Manders’ colocalization coefficient for the overlap between the *Pink1* mRNA and mitochondrial channel as in j. One-way ANOVA followed by Tukey’s post hoc test; n = 17-19. **l** Representative images of neurites displaying the PLA and mitotracker signal upon starvation (minus insulin, 2 h) and insulin (500 nM, 1 h) treatment. All data are expressed as mean ± SEM. All data points represent single cells coming from ≥3 biological replicates. Scale bars, 10 µm. Source data

One major signalling molecule absent in Hibernate E medium, but present in abundance in the neuronal supplement B27, is the peptide hormone insulin. Fittingly, activation of AKT downstream of IR signalling has already been shown to negatively regulate AMPK signalling^15^. We verified this negative regulation in neurons using lifetime imaging of a FRET-based AMPK activity sensor^25^. Indeed, if we added increasing amounts of insulin to Hibernate E medium, we observed a dose-dependent decrease in AMPK activity, indicated by an increase in lifetime of the FRET donor (Extended Data Fig. 3a). This effect was prevented by co-treatment with inhibitors directed against the IR (GSK1904529A), PI3K (Wortmannin) or AKT (AKT inhibitor VIII) (Extended Data Fig. 3b), corroborating that also in neurons insulin addition induces inhibition of AMPK activity via activation of PI3K and AKT. Hence, we tested whether insulin treatment per se affects the localization of *Pink1* mRNA at mitochondria. Addition of insulin decreased the recruitment of *Pink1* mRNA to mitochondria both in the soma and in neurites as visualized by MS2/PP7-based mRNA imaging (Fig. 2a–c). This effect was dependent on the activity of the IR (Fig. 2a–c), as well as on the activity of PI3K and AKT (Extended Data Fig. 3c,d), confirming the involvement of this signalling pathway in the regulation of mitochondrial *Pink1* mRNA association. This effect was also observed at lower insulin concentrations (Extended Data Fig. 3e) arguing that this scenario could happen under physiological conditions in the brain (low nanomolar concentrations)^26^. Furthermore, the reduction in mitochondrial localization of the *Pink1* mRNA upon insulin addition was not as complete (down to chance levels; Extended Data Fig. 3f) as seen with CC treatment (Extended Data Fig. 1c), which is to be expected upon physiological inhibition of AMPK. This effect of insulin and CC was not accompanied by any measurable defect in the percentage of moving mitochondria in neurites (Extended Data Fig. 3g,h) or their speed (Extended Data Fig. 3i), arguing that mitochondrial health was not affected by these treatments. As we have previously shown that *Pink1* mRNA association with mitochondria is dependent on the RNA-binding protein SYNJ2a^3^, we tested whether the AMPK-mediated regulation of *Pink1* mRNA localization is also dependent on SYNJ2a. Indeed, overexpression of a SYNJ2a version that is constitutively targeted to mitochondria by fusion with the transmembrane domain of SYNJ2BP (SYNJ2mito), prevented the insulin-mediated dissociation of *Pink1* mRNA from mitochondria (Extended Data Fig. 3j,k) in contrast to overexpression of a control protein (SNAPmito) fused to the transmembrane domain of SYNJ2BP. Overexpression of an RNA-binding-deficient SYNJ2mito VQL/AAA mutant^3^, however, did not retain *Pink1* mRNA at mitochondria upon insulin treatment (Extended Data Fig. 3j,k). This confirms that the insulin- and AMPK-mediated regulation of *Pink1* mRNA localization is dependent on SYNJ2a.

**Extended Data Fig. 4:**
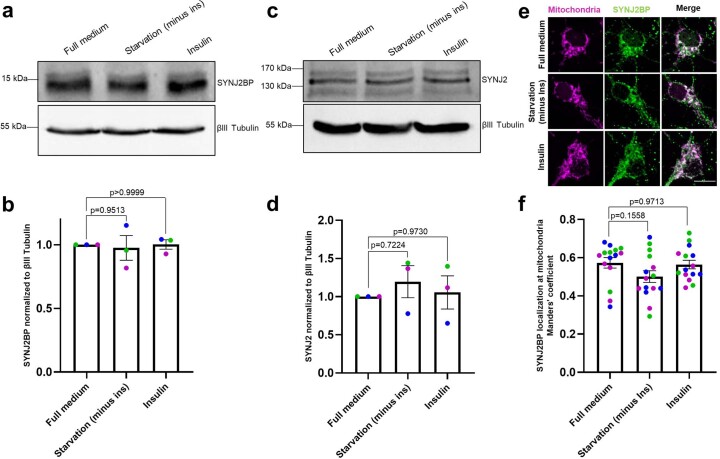
Insulin signalling does not affect the expression levels of SYNJ2BP and SYNJ2. **a** Representative immunoblot images of cortical neurons upon control (full medium), insulin starvation (2 h) or insulin addition (500 nM, 1 h). **b** Quantification of the SYNJ2BP protein bands normalized to the respective βIII tubulin bands as in a. One-way ANOVA followed by Tukey’s post hoc test; n = 3. **c** Representative immunoblot images of cortical neurons upon control (full medium), insulin starvation (2 h) or insulin addition (500 nM, 1 h). **d** Quantification of the SYNJ2 protein bands normalized to the respective βIII tubulin bands as in c. One-way ANOVA followed by Tukey’s post hoc test; n = 3. **e** Representative images of neurons overexpressing mito-meGFP neurons upon control (full medium), insulin starvation (2 h) or insulin addition (500 nM, 1 h) and stained with an antibody against SYNJ2BP. **f** Quantification of SYNJ2BP localization to mitochondria using the Manders’ colocalization coefficient as in e. One-way ANOVA followed by Tukey’s post hoc test; n = 15. All data are expressed as mean ± SEM. Data points represent biological replicates (**b,d**) or single cells coming from ≥3 biological replicates (**f**). Scale bar, 10 µm. Source data

Finally, we tested whether insulin, similar to CC, also reduced the interaction between SYNJ2 and SYNJ2BP using PLA. As before, we observed a significant increase in proximity when neurons were cultured for 2 h in medium lacking insulin (starvation). Addition of 500 nM insulin for 1 h completely reversed this effect, but only when IR signalling was not inhibited (Fig. 2d,e and Extended Data Fig. 3l). Notably, insulin withdrawal and addition of insulin had no effect on the expression levels of endogenous SYNJ2BP and SYNJ2 (Extended Data Fig. 4a–d) nor the mitochondrial localization of SYNJ2BP (Extended Data Fig. 4e,f). Together, these results suggest that physiological inhibition of AMPK via IR signalling and AKT activation controls mitochondrial localization of *Pink1* mRNA by regulating the interaction of the RNA-binding protein SYNJ2 with its mitochondrial anchor SYNJ2BP.

### AMPK phosphorylates SYNJ2BP in its PDZ domain

**Fig. 3:**
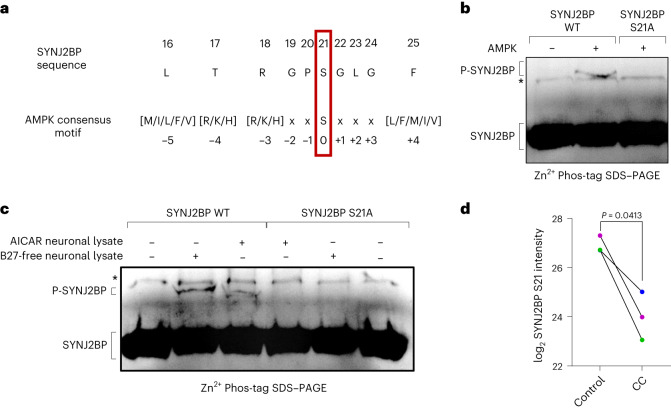
AMPK phosphorylates SYNJ2BP in its PDZ domain. **a**, Schematic showing the SYNJ2BP sequence around its proposed phosphorylation site S21 and the AMPK consensus motif. **b**, In vitro kinase assay analysed on a Zn^2+^-Phos-tag SDS–PAGE using recombinant AMPK as well as recombinant SYNJ2BP WT and S21A, respectively and decorated with a SYNJ2BP antibody. Note the appearance of a slower-migrating species only in the presence of AMPK and SYNJ2BP WT. The asterisk (*) denotes an unspecific band present in all samples. This is a representative blot of *n* > 3 experiments. **c**, In vitro phosphorylation assay as in **b**, using recombinant SYNJ2BP WT and S21A, respectively, treated with lysates from cortical neurons grown in vitro in the presence of AICAR or in the absence of the B27 supplement. Note the appearance of a slower-migrating species only in the presence of AICAR-treated or B27-free lysates and SYNJ2BP WT. The asterisk (*) denotes an unspecific band present in all samples. This is a representative blot of *n* > 3 experiments. **d**, Primary cortical neurons overexpressing myc-tagged SYNJ2BP WT by lentiviral transduction were cultured in insulin-free medium and treated with or without the AMPK inhibitor CC (20 µM, 2 h). The log_2_-transformed SYNJ2BP S21 intensity is shown upon phospho-peptide enrichment and LC–MS/MS analysis. Two-tailed Student’s *t*-test; *n* = 3. All data are expressed as mean ± s.e.m. All data points represent biological replicates. Source data

**Extended Data Fig. 5:**
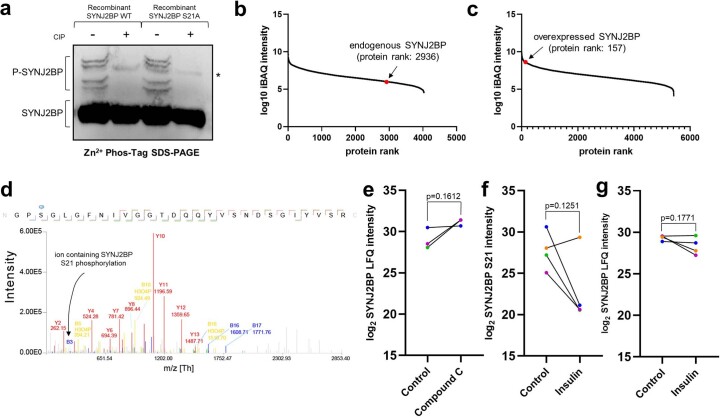
AMPK phosphorylates SYNJ2BP in its PDZ domain. **a** Dephosphorylation of recombinant SYNJ2BP WT and S21A, respectively, using CIP analysed on a Zn^2+^-Phos-Tag SDS–PAGE and decorated with a SYNJ2BP antibody. Note the appearance of slower migrating species that disappear upon addition of CIP indicating phosphorylated forms of SYNJ2BP. The asterisk (*) denotes an unspecific band that is not responsive to CIP treatment. This is a representative blot of n ≥ 3 experiments. **b-c** The number of quantified proteins upon LC–MS/MS analysis is plotted against the log10-transformed intensity-based absolute quantification (iBAQ) values. The iBAQ values represent an estimate of the molar abundance of proteins within the sample. Note, the protein rank of endogenous SYNJ2BP in cortical neurons (**b**) is relatively low, which can be improved by lentiviral overexpression of myc-tagged SYNJ2BP WT (**c**). **d** Annotated MS/MS spectrum of the peptide GPSGLGFNIVGGTDQQYVSNDSGIYVSR of lentivirally overexpressed myc-tagged SYNJ2BP in cortical neurons. Note, ion B3 represents the ion containing the phosphorylation at S21 of SYNJ2BP. **e** Log2-transformed LFQ intensities of SYNJ2BP upon LC–MS/MS analysis in primary cortical neurons that lentivirally overexpressed myc-tagged SYNJ2BP WT and were cultured in insulin-free medium and treated with or without the AMPK inhibitor CC (20 µM, 2 h). Two-tailed student’s t-test; n = 3. **f** Primary cortical neurons overexpressing myc-tagged SYNJ2BP WT by lentiviral transduction were cultured in insulin-free medium and treated with or without insulin (500 nM, 1 h). The log2-transformed SYNJ2BP S21 intensity is shown upon phospho-peptide enrichment and LC–MS/MS analysis. Two-tailed student’s t-test; n = 4. **g** Log2-transformed LFQ intensities of SYNJ2BP upon LC–MS/MS analysis in primary cortical neurons that lentivirally overexpress myc-tagged SYNJ2BP WT and were cultured in insulin-free medium and treated with or without insulin (500 nM, 1 h). Two-tailed student’s t-test; n = 4. All data are expressed as mean ± SEM, except **b** and **c**. All data points in **e**, **f**, and **g** represent biological replicates. Source data

Our observation that AMPK regulates the interaction between SYNJ2 and SYNJ2BP raised the question whether one of these proteins could be a direct substrate of AMPK. Notably, a phosphorylated peptide of SYNJ2BP matching the AMPK consensus motif^27^ (Fig. 3a) has been previously detected in high-throughput phospho-proteomics^28^. We therefore purified the cytosolic domain of rat SYNJ2BP from *Escherichia* *coli* and evaluated whether this protein could be phosphorylated in vitro by recombinant AMPK. To detect the phosphorylated protein, we employed the Phos-Tag approach^29^, which reduces the electrophoretic mobility of phosphorylated proteins. We observed that the purified SYNJ2BP was already phosphorylated in *E.* *coli*, which required pre-treatment of the protein with recombinant calf intestinal phosphatase (CIP), a broad specificity phosphatase to remove any unspecific phosphorylation, before performing the kinase assay using AMPK (Extended Data Fig. 5a). We detected a slower-migrating SYNJ2BP species upon addition of AMPK to the phosphorylation reaction, but this was prevented when the recombinant SYNJ2BP carried a mutation of the predicted phospho-site serine 21 to alanine (S21A; Fig. 3b). To test whether phosphorylation at this site responds to the physiological levels of AMPK activity present in neurons, we replaced the recombinant AMPK with lysates obtained from cortical neurons grown in vitro in the presence or absence of B27 (and therefore insulin), as well as lysates of neurons treated with AICAR to stimulate AMPK activity. Fittingly, we observed the slower-migrating, phosphorylated SYNJ2BP only upon B27 starvation or AMPK activation, but not when neurons were grown in insulin-containing medium or when the S21A mutant was used (Fig. 3c).

To test whether this phospho-site is observed in living neurons we employed phospho-peptide enrichment before mass spectrometry (phospho-MS); however, due to the low abundance of SYNJ2BP in cultured neurons (Extended Data Fig. 5b) we could not detect a phosphorylated peptide. We therefore overexpressed myc-tagged SYNJ2BP in neurons by lentiviral transduction, which increased its cellular abundance (Extended Data Fig. 5c) and finally allowed us to observe a phosphorylated peptide upon phospho-MS, indeed showcasing phosphorylation at serine 21 (S21) (Extended Data Fig. 5d). Fittingly, SYNJ2BP S21 phosphorylation was reduced in cortical neurons grown in insulin-free medium for 2 h and at the same time treated with the AMPK inhibitor CC (Fig. 3d), whereas the total levels of SYNJ2BP did not decrease (Extended Data Fig. 5e). Similarly, insulin treatment also reduced SYNJ2BP S21 phosphorylation within three out of four replicates (Extended Data Fig. 5f), whereas the total levels did not change (Extended Data Fig. 5g). Together, these results indicate that SYNJ2BP is a substrate of AMPK and its phosphorylation status is regulated by insulin signalling.

### SYNJ2BP phosphorylation regulates *Pink1* mRNA localization

**Fig. 4:**
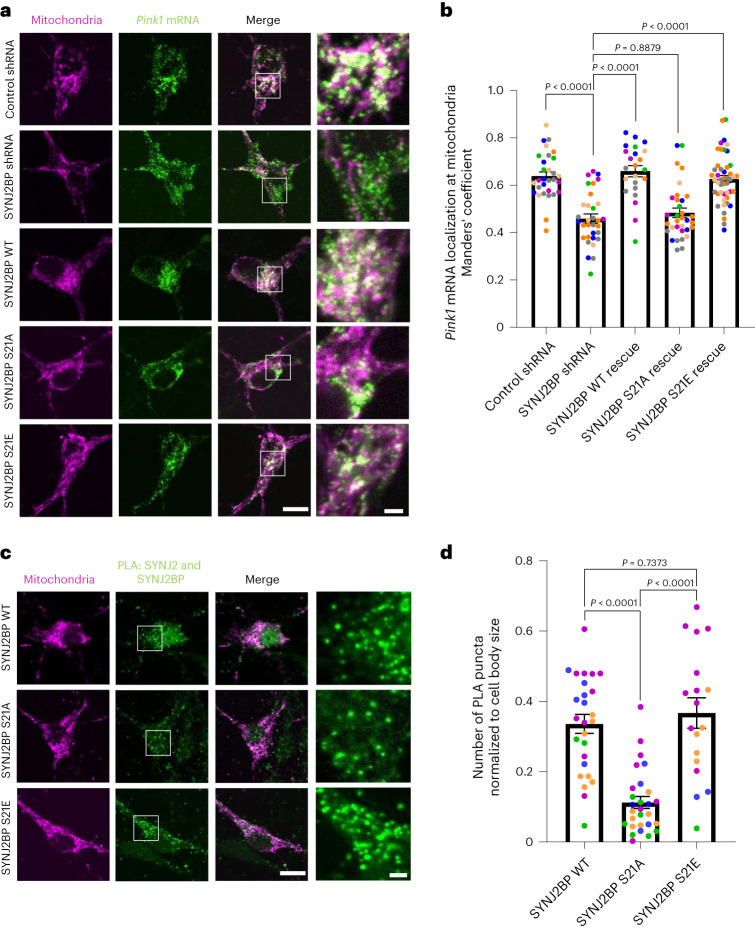
SYNJ2BP phosphorylation regulates *Pink1* mRNA localization to mitochondria. **a**, Representative images of *Pink1* mRNA visualized by the MS2/PP7-split-Venus method and mitoRaspberry upon control or SYNJ2BP shRNA expression combined with overexpression of shRNA-resistant SYNJ2BP WT, S21A or S21E in the soma. **b**, Quantification of the Manders’ colocalization coefficient for the overlap between the *Pink1* mRNA and mitochondrial channel as in **a**. One-way ANOVA followed by Tukey’s post hoc test; *n* = 23–46. **c**, Representative images of neuronal somata overexpressing mitoRaspberry as well as SYNJ2BP WT, S21A and S21E, respectively, displaying the PLA signal between SYNJ2BP and SYNJ2. **d**, Number of PLA puncta per soma of neurons normalized to the soma size as in **c**. One-way ANOVA followed by Tukey’s post hoc test; *n* = 18–28. All data are expressed as mean ± s.e.m. All data points represent single cells coming from at least three biological replicates. Scale bar, 10 µm; scale bars in insets, 2 µm. Source data

**Extended Data Fig. 6:**
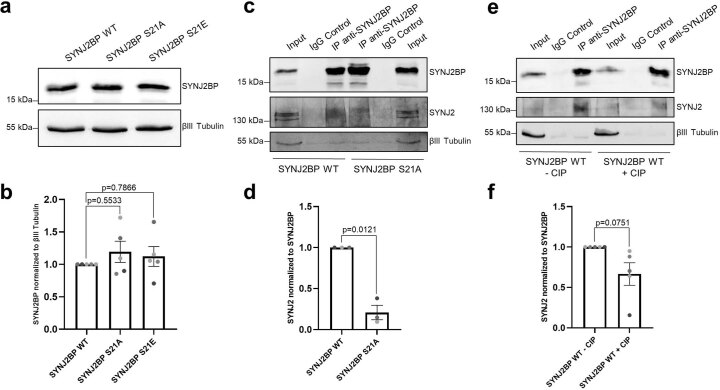
Expression levels of myc-tagged SYNJ2BP WT, S21A, and S21E constructs and Co-IP between SYNJ2BP and SYNJ2. **a** Representative immunoblot image of cortical neurons that lentivirally overexpress myc-tagged SYNJ2BP WT, S21A and S21E, respectively. **b** Quantification of the SYNJ2BP protein bands normalized to the respective βIII tubulin bands as in a. One-way ANOVA followed by Tukey’s post hoc test; n = 5. **c** Representative immunoblot image of SYNJ2BP immunoprecipitation using lysates of cortical neurons lentivirally overexpressing myc-tagged SYNJ2BP WT or S21A. Note, less SYNJ2 co-precipitated with SYNJ2BP S21A. **d** Quantification of the SYNJ2 protein bands normalized to the respective SYNJ2BP band as in c. Two-tailed Welch’s t-test; n = 3. **e** Representative immunoblot image of SYNJ2BP immunoprecipitation using lysates of cortical neurons lentivirally overexpressing myc-tagged SYNJ2BP WT treated with or without calf intestinal phosphatase (CIP). Note, less SYNJ2 co-precipitated with SYNJ2BP from lysates treated with CIP. **f** Quantification of the SYNJ2 protein bands normalized to the respective SYNJ2BP bands as well as the input band as in e. Two-tailed Welch’s t-test; n = 5. All data are expressed as mean ± SEM. All data points represent biological replicates. Source data

Our observation of AMPK-mediated phosphorylation at S21 in SYNJ2BP prompted us to investigate whether mutating this residue to alanine or to the phospho-mimetic amino acid glutamate (S21E) would replicate the effects of AMPK on *Pink1* mRNA localization visualized by MS2/PP7-split-Venus imaging. Indeed, while expression of an shRNA-resistant wild-type (WT) version of SYNJ2BP was fully able to rescue the loss of mitochondrial localization of *Pink1* mRNA upon SYNJ2BP knockdown, only the phospho-mimetic S21E, but not the phospho-ablative S21A mutant was able to rescue *Pink1* transcript localization (Fig. 4a,b). Notably, this was not due to reduced expression or stability of these rescue constructs (Extended Data Fig. 6a,b). This fully supports the model that AMPK-mediated phosphorylation at S21 of SYNJ2BP positively regulates *Pink1* mRNA recruitment to mitochondria.

To test whether the interaction of SYNJ2BP with SYNJ2 depends on its phosphorylation status, we immunoprecipitated virally overexpressed myc-tagged SYNJ2BP from cortical neurons grown in insulin-free medium for 2 h and determined the amount of co-isolated endogenous SYNJ2. We detected a specific interaction between the two proteins, as an abundant neuronal protein, βIII tubulin, was not co-isolated (Extended Data Fig. 6c,e). This interaction was diminished, when the phospho-ablative mutant of SYNJ2BP S21A was overexpressed (Extended Data Fig. 6c,d) or when the lysates were treated with CIP, a broad-range phosphatase to remove any phospho-sites of proteins in the lysate (Extended Data Fig. 6e,f). Moreover, overexpression of SYNJ2BP WT or the phospho-mimetic S21E mutant resulted in a significantly increased PLA signal with the endogenous SYNJ2 compared to overexpression of the phospho-ablative S21A mutant (Fig. 4c,d). Together, this shows that SYNJ2BP phosphorylation at S21 is a crucial prerequisite for efficient binding of SYNJ2 and hence regulates the localization of *Pink1* mRNA to mitochondria.

### Phospho-mimetic SYNJ2BP restores mitochondrial *Pink1* mRNA

**Fig. 5:**
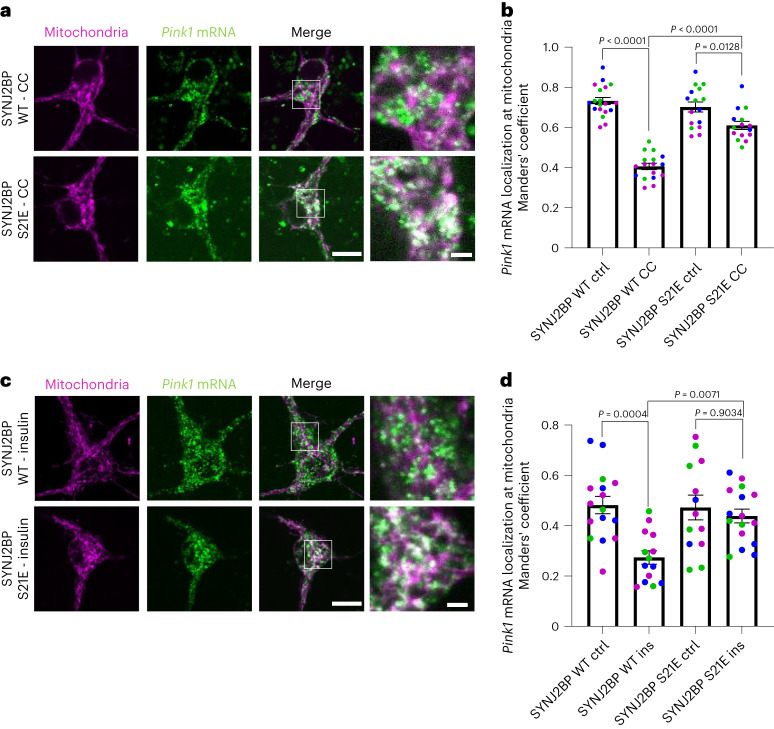
Phospho-mimetic SYNJ2BP restores mitochondrial *Pink1* mRNA localization upon AMPK inhibition. **a**, Representative images of *Pink1* mRNA visualized by the MS2/PP7-split-Venus method and mitoRaspberry upon CC (20 µM, 2 h) treatment combined with overexpression of SYNJ2BP WT and S21E, respectively. **b**, Quantification of the Manders’ colocalization coefficient for the overlap between the *Pink1* mRNA and mitochondrial channel in the soma of neurons overexpressing SYNJ2BP WT or S21E and treated with or without CC (20 µM, 2 h). One-way ANOVA followed by Tukey’s post hoc test; *n* = 16–19. **c**, Representative images of *Pink1* mRNA and mitoRaspberry upon insulin (500 nM, 1 h) treatment combined with overexpression of SYNJ2BP WT and S21E, respectively. **d**, Quantification of the Manders’ colocalization coefficient for the overlap between the *Pink1* mRNA and mitochondrial channel in the soma of neurons overexpressing SYNJ2BP WT or S21E and treated with or without insulin (500 nM, 1 h). One-way ANOVA followed by Tukey’s post hoc test; *n* = 13–16. All data are expressed as mean ± s.e.m. All data points represent single cells coming from at least three biological replicates. Scale bars, 10 µm. Source data

**Extended Data Fig. 7:**
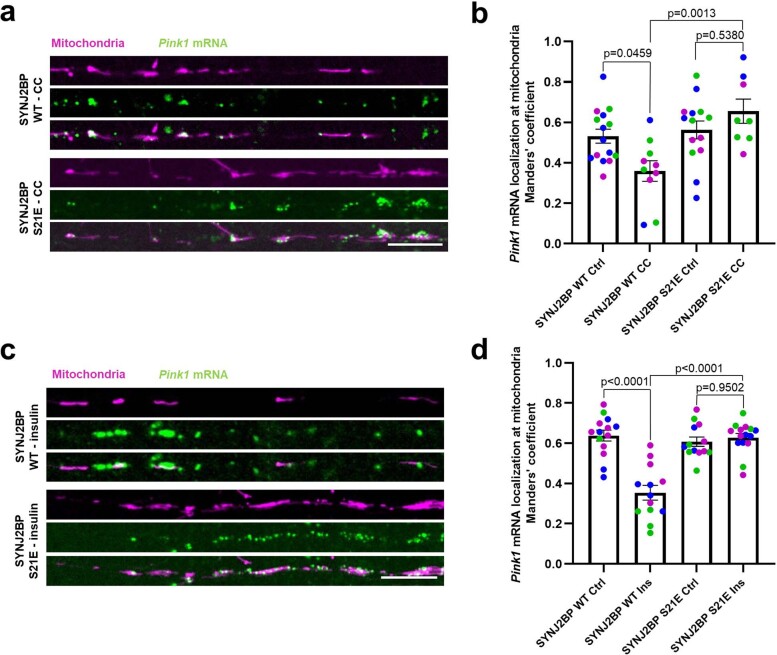
Phospho-mimetic SYNJ2BP restores mitochondrial Pink1 mRNA localization upon AMPK inhibition in neurites. **a** Representative images of *Pink1* mRNA visualized by the MS2/PP7-split Venus method and mitoRaspberry in neurites upon CC (20 µM, 2 h) treatment combined with overexpression of SYNJ2BP WT or S21E. **b** Quantification of the Manders’ colocalization coefficient for the overlap between the *Pink1* mRNA and mitochondrial channel in neurites overexpressing SYNJ2BP WT or S21E and treated with or without CC (20 µM, 2 h). One-way ANOVA followed by Tukey’s post hoc test; n = 8-15. **c** Representative images of *Pink1* mRNA and mitoRaspberry in neurites upon insulin (500 nM, 1 h) treatment combined with overexpression of SYNJ2BP WT or S21E. **d** Quantification of the Manders’ colocalization coefficient for the overlap between the *Pink1* mRNA and mitochondrial channel in neurites overexpressing SYNJ2BP WT or S21E and treated with or without insulin (500 nM, 1 h). One-way ANOVA followed by Tukey’s post hoc test; n = 13-15. All data are expressed as mean ± SEM. All data points represent single cells coming from ≥3 biological replicates. Scale bars, 10 µm. Source data

As we observed that the phospho-mimetic S21E mutant of SYNJ2BP was sufficient to restore *Pink1* mRNA localization (Fig. 4a,b), we wondered whether expression of this mutant could overcome the effects of AMPK inhibition. Therefore, we tested whether SYNJ2BP S21E overexpression would prevent the CC-mediated dissociation of *Pink1* mRNA from mitochondria, using the MS2/PP7-split-Venus method. Indeed, the loss of colocalization induced by CC treatment was greatly diminished both in the soma and in neurites when the phospho-mimetic S21E mutant was overexpressed instead of WT SYNJ2BP (Fig. 5a,b and Extended Data Fig. 7a,b). Similarly, the effect of insulin addition was abolished upon expression of SYNJ2BP S21E (Fig. 5c,d and Extended Data Fig. 7c,d), fully supporting the model that phosphorylation of SYNJ2BP S21 underlies the regulation of *Pink1* mRNA localization by insulin and AMPK signalling.

### Insulin supports PINK1 activation and mitophagy

**Fig. 6:**
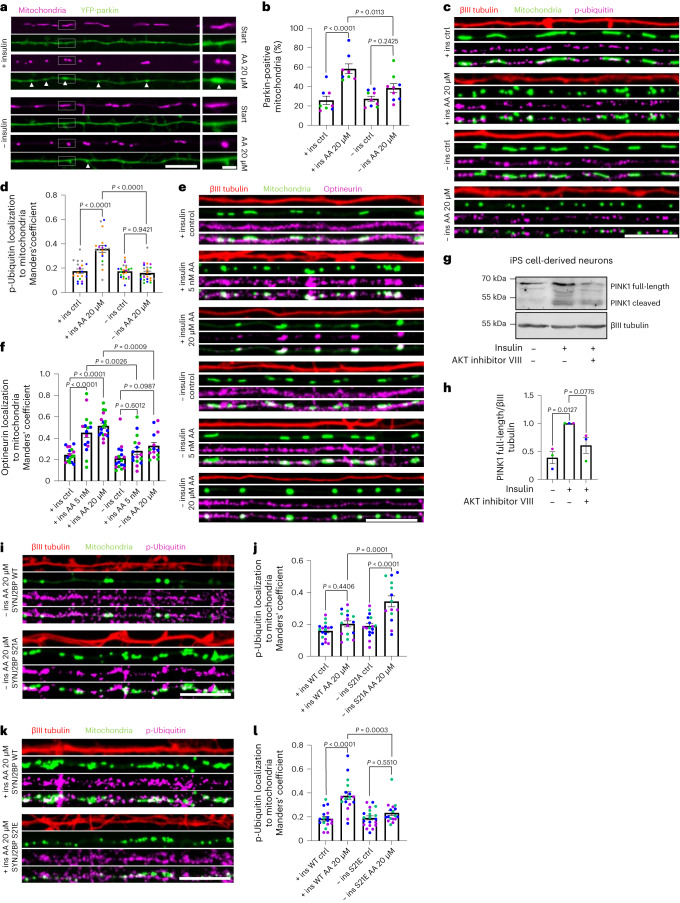
Insulin supports PINK1 activation and mitophagy. **a**, Representative images of neurites overexpressing YFP-Parkin and mitoRaspberry cultured and treated as indicated. The white arrowheads indicate Parkin recruitment to mitochondria. **b**, Quantification of mitochondrial Parkin recruitment as in **a**. One-way ANOVA followed by Tukey’s post hoc test; *n* = 8–9. **c**, Representative images of neurites overexpressing mito-meGFP cultured and treated as indicated and stained for p-ubiquitin (S65) and βIII tubulin. **d**, Quantification of p-ubiquitin (S65) localization to mitochondria using the Manders’ colocalization coefficient as in **c**. One-way ANOVA followed by Tukey’s post hoc test; *n* = 19–24. **e**, Representative images of neurites overexpressing mito-meGFP cultured and treated as indicated and stained for optineurin and βIII tubulin. **f**, Quantification of optineurin recruitment to mitochondria using the Manders’ colocalization coefficient as in **e**. One-way ANOVA followed by Tukey’s post hoc test; *n* = 14–18. **g**, Representative immunoblot image of human iPS cell-derived cortical neurons cultured and treated as indicated for 2 h. **h**, Quantification of the full-length PINK1 protein bands normalized to the βIII tubulin signal as in **g**. One-way ANOVA followed by Tukey’s post hoc test; *n* = 3. **i**, Representative images of neurites overexpressing mito-meGFP as well as SYNJ2BP WT or S21A cultured and treated as indicated and stained for p-ubiquitin (S65) and βIII tubulin. **j**, Quantification of p-ubiquitin (S65) localization to mitochondria using the Manders’ colocalization coefficient. One-way ANOVA followed by Tukey’s post hoc test; *n* = 14–17. **k**, Representative images of neurites overexpressing mito-meGFP as well as SYNJ2BP WT or S21E cultured and treated as indicated and stained for p-ubiquitin (S65) and βIII tubulin. **l**, Quantification of p-ubiquitin (S65) localization to mitochondria using the Manders’ colocalization coefficient. One-way ANOVA followed by Tukey’s post hoc test; *n* = 17–20. All data are expressed as mean ± s.e.m. Data points represent biological replicates (**h**) or single cells coming from at least three biological replicates (**b**,**d**,**f**,**j**,**I**). Scale bars, 10 µm. Source data

**Extended Data Fig. 8:**
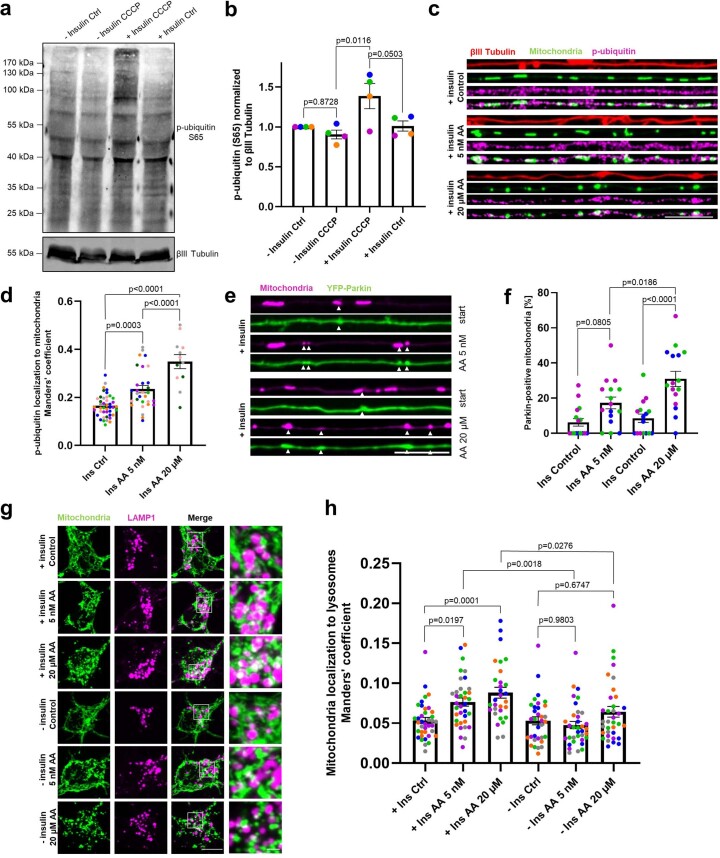
Mild damage induces PINK1/Parkin-dependent mitophagy. **a** Representative immunoblot image of human iPSC-derived neurons cultured with or without insulin overnight and treated with or without CCCP (20 µM, 2 h). **b** Quantification of p-ubiquitin levels normalized to the βIII tubulin signal as in a. One-way ANOVA followed by Tukey’s post hoc test; n = 4. **c** Representative images of neurites overexpressing mito-meGFP cultured in the presence of insulin overnight prior to treatment with 5 nM or 20 µM AA (45 min) and stained with antibodies against p-ubiquitin (S65) and βIII tubulin. **d** Quantification of p-ubiquitin (S65) localization to mitochondria using the Manders’ colocalization coefficient as in c. One-way ANOVA followed by Tukey’s post hoc test; n = 12-42. **e** Representative images of neurites overexpressing YFP-Parkin and mitoRaspberry cultured in the presence of insulin overnight prior to treatment with 5 nM or 20 µM AA. The white arrowheads indicate Parkin recruitment to mitochondria monitored with live-cell imaging before (start) and after 30 min of AA addition. **f** Quantification of mitochondria colocalizing with Parkin before and after AA treatment in presence of insulin as in e. One-way ANOVA followed by Tukey’s post hoc test; n = 16. **g** Representative image of neurons overexpressing mito-meGFP and LAMP1-mCherry cultured in the presence or absence of insulin overnight prior to treatment with or without 5 nM or 20 µM AA (45 min). **h** Quantification of mitochondrial localization to lysosomes using the Manders’ colocalization coefficient as in g. One-way ANOVA followed by Tukey’s post hoc test; n = 30-38. All data are expressed as mean ± SEM. Data points represent biological replicates (**b**) or single cells coming from ≥3 biological replicates (**d,f,h**). Scale bars, 10 µm for whole soma and neurites, 2 µm for insets. Source data

The main function of PINK1 is the tagging of damaged mitochondria with phosphorylated ubiquitin, which is added by the recruitment of the E3 ubiquitin ligase Parkin to the organelle. Therefore, we analysed whether recruitment of Parkin to damaged mitochondria was affected by the presence of insulin using live-cell microscopy. We took advantage of the decreased mitochondrial density in neurites compared to the soma to allow for the visualization of YFP-Parkin enrichment on mitochondria upon mitophagy induction. Unexpectedly, we observed that recruitment of Parkin to mitochondria upon mitochondrial damage with complex III inhibitor antimycin A (AA; 20 µM) was decreased in neurites grown in the absence of insulin overnight (Fig. 6a,b), despite increased mRNA tethering (Fig. 2a–c). This suggests that mRNA tethering and mitophagy induction might be reciprocally regulated. To further test PINK1 activity, we probed the generation of phospho-ubiquitin (p-ubiquitin) at mitochondria using a phospho-specific antibody in primary mouse hippocampal neurons, cultured either in the presence or absence of insulin overnight. We performed the analysis in neurites whose integrity was confirmed by βIII tubulin staining. While the baseline levels of mitochondrial p-ubiquitin remained unchanged, neurons grown in insulin-free medium were unable to boost mitochondrial p-ubiquitination upon induction of mitophagy with 20 µM AA (Fig. 6c,d). We confirmed this observation by immunoblotting in human induced pluripotent stem (iPS) cell-derived neurons. Neurons cultured in insulin-free medium exhibited a significant reduction in p-ubiquitin levels upon induction of mitochondrial damage using 20 µM CCCP (Extended Data Fig. 8a,b). The lack of mitochondrial p-ubiquitination in the absence of insulin is consistent with reduced Parkin recruitment.

To further understand the role of insulin signalling in the recruitment of mitochondria into autophagosomes, we analysed AA-induced mitochondrial recruitment of optineurin (Fig. 6e,f), one of the main autophagy receptors in PINK1/Parkin-dependent mitophagy^11^. As optineurin may also be involved in Parkin-independent forms of mitophagy^30^, especially at high damage, we included a lower concentration of AA (5 nM), which is sufficient to induce p-ubiquitination (Extended Data Fig. 8c,d) and mild Parkin recruitment (Extended Data Fig. 8e,f) as well as targeting of mitochondria to the lysosome as measured by colocalization between mitochondria and LAMP1-positive lysosomes (Extended Data Fig. 8g,h). Both upon high (20 µM) and low (5 nM) AA concentrations, optineurin was recruited to damaged mitochondria, but only in the presence of insulin (Fig. 6e,f). Similarly, colocalization of mitochondria and LAMP1-positive lysosomes following AA addition only occurred, when neurons were cultured with insulin overnight (Extended Data Fig. 8g,h). Notably, while some colocalization between intact mitochondria and lysosomes was always observed, only in the presence of insulin and upon mitochondrial damage we observed a complete overlap of the two signals (Extended Data Fig. 8g, insets). This further supports the positive effect of insulin on mitophagy.

**Extended Data Fig. 9:**
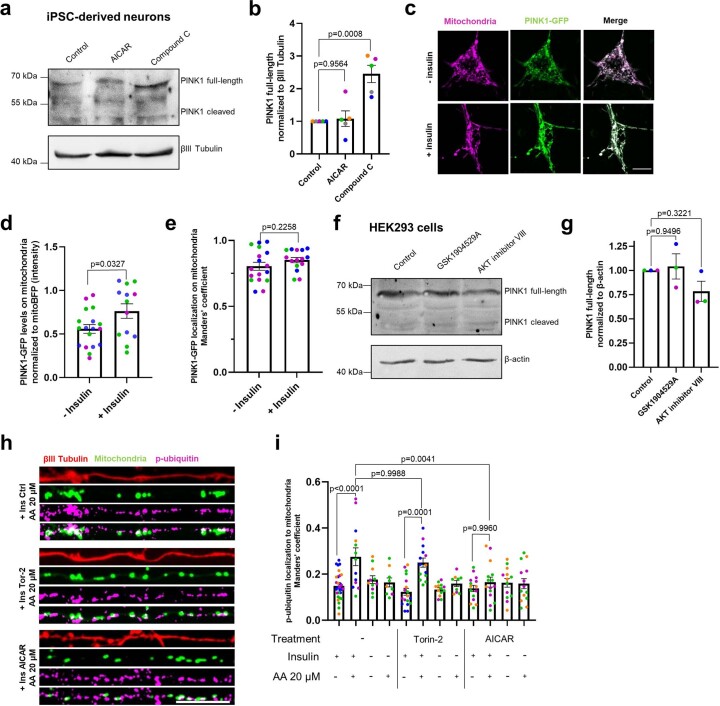
Insulin-regulated PINK1 expression is neuron-specific and mTORC1-independent. **a** Representative immunoblot image of human iPSC-derived neurons upon control, AICAR (1 mM, 2 h) or CC (20 µM, 2 h) treatment. **b** Quantification of the full-length PINK1 protein bands normalized to the βIII tubulin signal as in a. One-way ANOVA followed by Tukey’s post hoc test; n = 5. **c** Representative images of neurons overexpressing PINK1-GFP and mito-BFP cultured in the presence or absence of insulin for 2 h. **d** Quantification of the mitochondrial PINK1-GFP intensity normalized to the mito-BFP intensity as in c. Two-tailed student’s t-test; n = 13-18. **e** Quantification of the Manders’ colocalization coefficient for the overlap between the PINK1-GFP and the mitochondrial channel as in d. Two-tailed student’s t-test; n = 15-17. **f** Representative immunoblot image of HEK293 cells upon vehicle control, IR inhibitor GSK1904529A (1 µM, 2 h) or AKT inhibitor VIII (10 µM, 2 h) treatment. **g** Quantification of the full-length PINK1 protein bands normalized to the β-actin signal as in f. One-way ANOVA followed by Tukey’s post hoc test; n = 3. **h** Representative images of neurites overexpressing mito-meGFP cultured in the presence of insulin as well as Torin-2 (10 nM, 30 min) or AICAR (1 mM, 2 h) prior to treatment with 20 µM AA and stained with antibodies against p-ubiquitin (S65) and βIII tubulin. **i** Quantification of p-ubiquitin (S65) localization to mitochondria using the Manders’ colocalization coefficient. One-way ANOVA followed by Tukey’s post hoc test; n = 12-19. All data are expressed as mean ± SEM. Data points represent biological replicates (**b, g**) or single cells coming from ≥3 biological replicates (**d, e, i**). Scale bars, 10 µm. Source data

To understand the opposing effects of insulin on mRNA tethering and mitophagy induction, we sought to investigate its effect on PINK1 expression levels. We took advantage of a PINK1 antibody recognizing the human PINK1 protein and compared the levels of PINK1 protein in human iPS cell-derived cortical neurons cultured in medium either supplemented with or without insulin. Two hours of insulin withdrawal reduced all major PINK1 protein species, as did AKT inhibition (Fig. 6g,h), in accordance with the very short half-life of the protein^5^ and with reduced mitophagic capacity under these conditions (Fig. 6a–f). In line with this, AMPK inhibition using CC resulted in a significant increase of PINK1 protein levels, whereas AICAR treatment had no effect (Extended Data Fig. 9a,b). As a complementary approach, we assessed PINK1 levels on mitochondria in living mouse hippocampal neurons by overexpressing GFP-tagged PINK1 and also observed a reduction in intensity upon insulin withdrawal for 2 h (Extended Data Fig. 9c,d). Of note, this did not coincide with a reduction in the colocalization of PINK1 with mitochondria, suggesting that this effect is not due to altered mitochondrial targeting of PINK1 (Extended Data Fig. 9e). In HEK293 cells, inhibition of insulin signalling did not result in lower PINK1 expression (Extended Data Fig. 9f,g), suggesting that this is a neuron-specific mechanism and therefore might be tied to the neuron-specific tethering of the *Pink1* mRNA to mitochondria^3^. Hence, the mitophagy defect upon insulin withdrawal (Fig. 6a–f) could be explained by a reduced availability of the *Pink1* transcript due to its mitochondrial association (Fig. 2a–c).

This insulin-mediated boost in PINK1 availability/activity did not depend on general translational regulation by mammalian target of rapamycin (mTOR) signalling, as mTOR inhibition using Torin-2 still resulted in mitochondrial p-ubiquitination upon AA treatment but could be suppressed by addition of the AMPK activator AICAR (Extended Data Fig. 9h,i). This suggested that the activity of PINK1 is regulated by AMPK downstream of insulin signalling independently of mTOR signalling, and hence could be regulated at the level of SYNJ2BP phosphorylation. We therefore tested whether mutating SYNJ2BP S21 would be sufficient to prevent the block in PINK1 activity seen upon insulin withdrawal. Indeed, expression of the phospho-ablative SYNJ2BP S21A but not WT prevented the block in PINK1 activity in medium lacking insulin (Fig. 6i,j). This is consistent with the model that sequestering of the mRNA at mitochondria is responsible for the lack of PINK1 production, as the S21A mutant is unable to efficiently recruit the *Pink1* mRNA to mitochondria (Fig. 4a,b). Concurrently, in insulin-containing medium, PINK1 activation can occur in the presence of WT SYNJ2BP but is blocked in the presence of the phospho-mimetic S21E mutant (Fig. 6k,l), which retains the mRNA at mitochondria even in the presence of insulin (Fig. 5c,d). Thus, insulin-mediated uncoupling of the *Pink1* mRNA from mitochondria is a prerequisite for PINK1 activation and efficient induction of PINK1/Parkin-dependent mitophagy.

### ApoE4 inhibits insulin-regulated *Pink1* mRNA localization

**Fig. 7:**
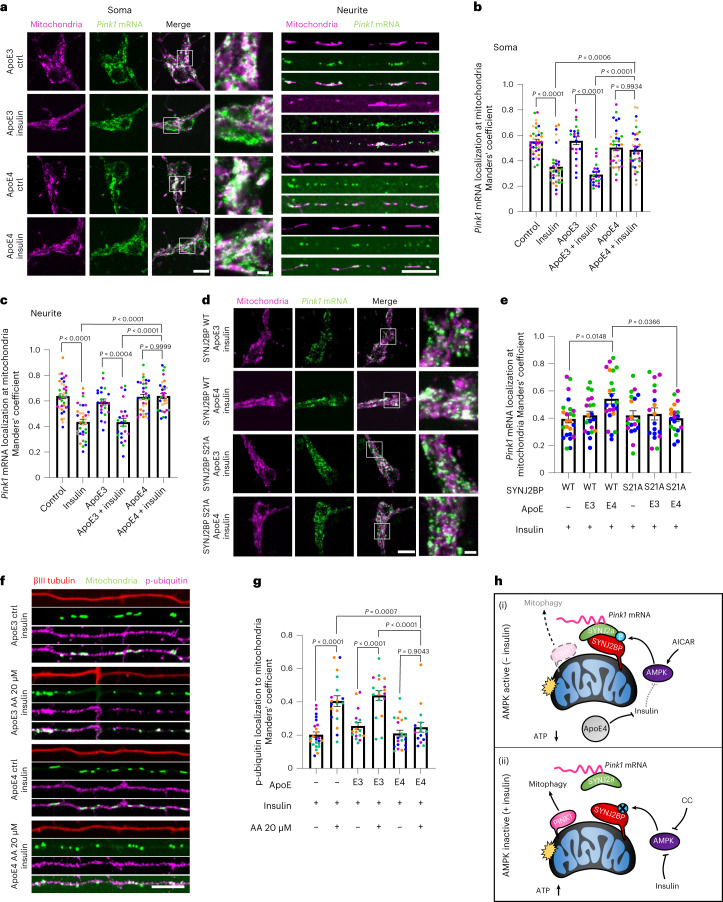
ApoE4 inhibits insulin-regulated *Pink1* mRNA localization and PINK1 activation. **a**, Representative images of *Pink1* mRNA visualized by the MS2/PP7-split-Venus method and mitoRaspberry with and without insulin treatment in the presence of ApoE3 or ApoE4. **b**, Quantification of *Pink1* mRNA mitochondrial localization using Manders’ colocalization coefficient in the soma. One-way ANOVA followed by Tukey’s post hoc test; *n* = 21–30. **c**, Quantification as in **b** for neurites. One-way ANOVA followed by Tukey’s post hoc test; *n* = 22–37. **d**, Representative images of *Pink1* mRNA visualized by the MS2/PP7-split-Venus method and mitoRaspberry upon overnight treatment with insulin as well as ApoE3 and ApoE4, combined with overexpression of SYNJ2BP WT and S21A as indicated. **e**, Quantification of *Pink1* mRNA mitochondrial localization using Manders’ colocalization coefficient as in **d**. One-way ANOVA followed by Tukey’s post hoc test, *n* = 18–24. **f**, Representative images of neurites overexpressing mito-meGFP cultured in the presence of insulin as well as ApoE3 and ApoE4 overnight, treated as indicated and stained for p-ubiquitin (S65) and βIII tubulin. **g**, Quantification of p-ubiquitin (S65) localization to mitochondria using the Manders’ colocalization coefficient as in **f**. One-way ANOVA followed by Tukey’s post hoc test; *n* = 16–24. **h**, Model of insulin- and AMPK-mediated regulation of mitochondrial *Pink1* mRNA localization. (i) When insulin levels are low and AMPK is active, *Pink1* mRNA is localized to mitochondria via SYNJ2BP and SYNJ2a. As a result, PINK1 protein levels are reduced and less mitophagy is observed. ApoE4 mimics this state by inhibiting the insulin-mediated effects. (ii) When insulin levels are high and AMPK is less active, the interaction between SYNJ2BP and SYNJ2a is reduced and less *Pink1* mRNA is tethered to mitochondria. As a result, PINK1 protein levels are increased and mitophagy is facilitated. All data are expressed as mean ± s.e.m. All data points represent single cells coming from at least three biological replicates. Scale bars, 10 µm; scale bar in insets, 2 µm. Source data

**Extended Data Fig. 10:**
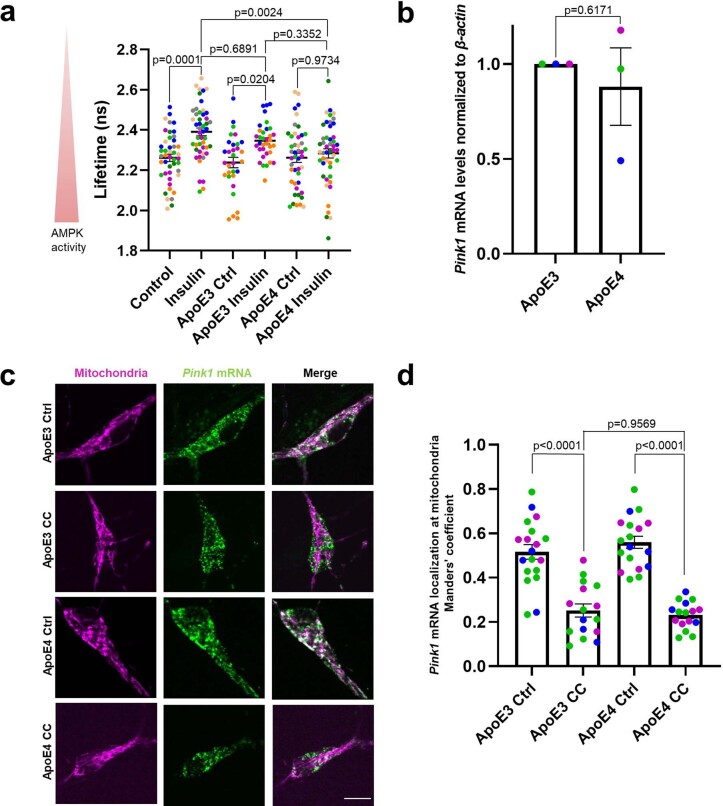
ApoE4 inhibits insulin- but not CC-mediated effects. **a** Quantification of fluorescent lifetime imaging of the FRET-based AMPK activity sensor in neurons treated with and without insulin (500 nM, 1 h) in the presence of ApoE3 (50 nM) or ApoE4 (50 nM) overnight. One-way ANOVA followed by Tukey’s post hoc test; n = 31-47. **b** RT-qPCR of *Pink1* transcript levels normalized to β-*actin* from primary cortical neurons treated with ApoE3 or ApoE4 (50 nM, overnight). Two-tailed Welch’s t-test; n = 3. **c** Representative images of *Pink1* mRNA visualized by the MS2/PP7-split Venus method and mitoRaspberry with or without CC (20 µM, 2 h) treatment in the presence of ApoE3 (50 nM) or ApoE4 (50 nM) overnight, respectively. **d** Quantification of the Manders’ colocalization coefficient for the overlap between the *Pink1* mRNA and mitochondrial channel as in c. One-way ANOVA followed by Tukey’s post hoc test; n = 11-14. All data are expressed as mean ± SEM. All data points represent biological replicates (**b**) or single cells coming from ≥3 biological replicates (**a**,**d**). Scale bars, 10 µm. Source data

Brain insulin resistance is believed to contribute to the observed metabolic changes in AD^17^, yet no direct connection between insulin resistance and mitochondrial dysfunction has been shown in neurons. Our results suggest that defective insulin signalling will have direct consequences on *Pink1* mRNA localization and downstream mitochondrial quality. Therefore, we tested whether *Pink1* mRNA localization is altered in an in vitro model of insulin resistance by application of human ApoE4, a highly prevalent AD risk factor^18^. ApoE4 application to cultured neurons has previously been shown to blunt IR signalling by sequestration of the IR into endosomes^19^. We confirmed that application of ApoE4 also prevented the insulin-induced decrease in AMPK activity, using lifetime measurement of the FRET-based AMPK activity sensor. Indeed, neurons treated with ApoE4 overnight were unable to modulate AMPK activity in response to insulin application (Extended Data Fig. 10a). This effect was specific to ApoE4 as treatment with its homologue ApoE3 did not alter the response to insulin (Extended Data Fig. 10a). Furthermore, ApoE4 treatment prevented the insulin-mediated decline in mitochondrial *Pink1* mRNA localization both in the soma and in neurites as visualized with the MS2/PP7-split-Venus system. ApoE3 exposure, on the other hand, did not affect the insulin-induced impact on *Pink1* mRNA localization (Fig. 7a–c). Of note, the effect of ApoE4 on mitochondrial *Pink1* mRNA association was not due to changes in *Pink1* mRNA levels (Extended Data Fig. 10b). In contrast to insulin, CC-induced loss of mitochondrial *Pink1* mRNA localization was not affected by ApoE4 treatment (Extended Data Fig. 10c,d), suggesting that the effect of ApoE4 on *Pink1* mRNA localization is indeed upstream of AMPK-mediated phosphorylation of SYNJ2BP. Therefore, we tested whether overexpression of the phospho-ablative SYNJ2BP S21A mutant could prevent the ApoE4 effect in the presence of insulin. Indeed, mitochondrial *Pink1* mRNA localization was greatly diminished upon insulin and ApoE4 treatment, when the phospho-ablative S21A mutant was overexpressed instead of WT SYNJ2BP (Fig. 7d,e). As we have seen that untethering of the *Pink1* mRNA from mitochondria is required for full PINK1 activity, we tested whether ApoE4 application inhibited the accumulation of p-ubiquitin on mitochondria upon mitochondrial intoxication with AA. In insulin-containing medium, AA induced mitochondrial p-ubiquitin levels in neurites, but not when the cells were treated with ApoE4 overnight (Fig. 7f,g). Together, these data suggest that dysregulation of insulin- and AMPK-mediated *Pink1* mRNA localization and PINK1 activity are contributing to mitochondrial dysfunction under pathological conditions modelling insulin resistance in vitro (Fig. 7h). This provides a mechanistic connection between these two epiphenomena commonly seen in AD.

## Discussion

Our study identifies a metabolic control of *Pink1* mRNA localization in response to activation of IR signalling and downstream inhibition of AMPK. Upon insulin withdrawal, AMPK-mediated phosphorylation of the mitochondrial outer membrane protein SYNJ2BP increases its interaction with the RNA-binding protein SYNJ2 as well as the mitochondrial localization of *Pink1* mRNA (Figs. 4 and 5). This tight association allows efficient transport of the transcript into neurites via mitochondrial hitchhiking^3^. Fitting to its role as a master regulator of cellular metabolism, AMPK is known to impact several aspects of mitochondrial biology, ranging from activation of mitochondrial fission to suppression of retrograde transport and synaptic anchoring^21,31,32^. These mechanisms serve to increase mitochondrial presence in axons that undergo shortage of ATP due to synaptic activity. Our results predict that mitochondrial hitchhiking will be increased under these conditions, while at the same time local PINK1 availability will be reduced (Fig. 6). This limits the amount of local mitophagy, which further serves to increase the local mitochondrial content, at the expense of retaining potentially damaged organelles. Local biogenesis of PINK1 in neurons is then activated by untethering of the *Pink1* transcript from mitochondria and localization of the transcript to potentially translationally active late endosomes (Extended Data Fig. 1p,q). Further research using for example triple labelling of these organelles together with the *Pink1* mRNA reporter will have to clarify the fate of the *Pink1* mRNA at late endosomes. The dissociation of *Pink1* mRNA from mitochondria occurs during times of glucose abundance, as signalled by the presence of insulin and inhibition of AMPK signalling (Figs. 1 and 2). During these times, neurons may generate ATP through glycolysis as well as via mitochondrial OXPHOS^33^, reducing the absolute necessity of mitochondria and opening a window for mitochondrial quality control via PINK1.

In our model, neuronal PINK1-dependent mitophagy would be regulated oppositely to bulk autophagy, which increases during fasting via the activation of AMPK and inhibition of mTOR signalling^34^. This may be necessary in the morphologically complex environment of neurons, where mitostasis is particularly challenging^1^. Removal of a damaged organelle resident at a particular synapse will alter the local calcium dynamics^35^ and local mitophagy may therefore present a last resort that will only be activated if the mitochondrion is severely damaged. In line with this, PINK1/Parkin-mediated mitophagy is not observed under basal conditions in neurons^36^. Furthermore, although starvation reliably represses mTOR signalling in neurons, this does not lead to increased autophagy in cultured neurons^37^, which is consistent with our observations. Of note, a very recent study in non-neuronal cells demonstrated that during starvation, AMPK-mediated phosphorylation of ULK1 leads to the inhibition of ULK1 activity resulting in suppression of autophagy initiation. This finding challenges the current understanding and suggests that the connection between AMPK and autophagy is more complex than previously believed^38^. When faced with energy shortages, the cell may prioritize other vital processes over autophagy, as autophagy itself is energy-demanding. As suggested by our findings, this initial inhibition of autophagy and mitophagy mediated by AMPK might be particularly important in neurons considering their high energy demands. Inhibition of mTOR signalling also leads to repression of bulk translation, which is thought to decrease the energetic needs of the cell. Our study now adds an AMPK-dependent parallel pathway that attenuates local PINK1 synthesis in neurons. Besides its role in mitophagy, PINK1 has also been described to positively stimulate the translation of other mitochondria-associated mRNAs encoding mitochondrial OXPHOS proteins^39^. Restriction of PINK1 biogenesis to times of energy abundance may in turn also synchronize the biogenesis of several mitochondria-associated transcripts and limit energy-consuming local translation during ATP-limiting conditions. Most likely, other SYNJ2 substrates, such as *Atp5f1b*, might also be regulated in a similar fashion (Extended Data Fig. 1h,i), adding to the restriction of mitochondrial biogenesis under unfavourable conditions. Our results are in line with previous studies that connect PINK1-dependent mitophagy in neurons with the activity of the AKT kinase^40^. As this PI(3,4,5)P_3_ (PIP_3_)-activated kinase is a common downstream effector of several growth factor receptors, it is interesting to speculate that similar mechanisms also regulate *Pink1* mRNA localization upon signalling by other growth factors using this pathway. Of note, PINK1 itself has been suggested to positively regulate the levels of PIP_3_, which promotes AKT activity^41^. This could build a positive feedback loop that ensures efficient AKT activity locally to inhibit AMPK and release more *Pink1* mRNA from its mitochondrial sequestration to amplify local PINK1 activity and mitophagy.

It remains to be determined whether other mitophagy pathways, such as Parkin-independent mechanisms^42,43^, are also actively upregulated to balance the reduced activity of the PINK1/Parkin-dependent pathway during energy-limiting conditions. One potential candidate is FUNDC1-mediated mitophagy. AMPK signalling activates the general autophagosome machinery, including direct phosphorylation of the kinase ULK1 as part of the autophagy pre-initiation complex^44^. AMPK-phosphorylated ULK1 subsequently translocates to mitochondria and can phosphorylate and activate FUNDC1 (ref. ^45^). This may represent one of the alternative delivery routes of mitochondria to the autophagolysosome under energy-limited conditions. Also, Parkin has been shown to be phosphorylated by cytosolic ULK1 (ref. ^46^), which promotes the ability of PINK1 to activate Parkin. Nevertheless, it remains to be determined whether ULK1-mediated phosphorylation of Parkin or FUNDC1 contributes to mitophagy in neurons.

The insulin-mediated regulation of *Pink1* mRNA tethering to mitochondria may also serve to resolve some of the controversial results in the field. Low doses of AA have been shown to be insufficient to elicit Parkin recruitment to mitochondria^47^ and, notably, the hippocampal mouse neurons used in this study were imaged in buffer not containing any growth factors. Also, in iPS cell-derived neurons, differences in the efficacy of PINK1/Parkin-mediated mitophagy have been observed; while some studies were able to detect Parkin recruitment to mitochondria, others have argued that this pathway is non-functional in human iPS cell-derived neurons^48^. Our results now suggest that differential media compositions may underlie some of the observed variability. How much this also pertains to mitophagy in vivo is one of the limitations of our study. All experiments were performed in neurons cultured in vitro, but the metabolic environment of the intact brain involves a much more complex interplay of different cell types and hormonal regulations^16^. It will be interesting to follow up the regulation of mitophagy by insulin signalling in vivo, to untangle the cell-autonomous regulation of PINK1 biogenesis in neurons from the effect that altered insulin signalling has in the context of brain slices and intact organisms.

While we have focused our studies on neuronal cells, SYNJ2BP phosphorylation is not limited to neurons, as it was originally detected in large scale proteomics of breast cancer tissue^28^; however, *Pink1* mRNA tethering is restricted to cells that express SYNJ2a^3^. In line with this, HEK293 cells were not affected in their ability to express PINK1 in the absence of insulin signalling (Extended Data Fig. 9f,g). Notably, the expression of SYNJ2 is upregulated in human breast cancer tissue^49^. This suggests that SYNJ2 expression may coincide with phosphorylation of SYNJ2BP in cancer. It will be interesting to test whether mitophagy is similarly regulated under these pathological conditions as under physiological conditions in neurons.

Insulin resistance is frequently observed in AD pathology and altered brain glucose metabolism often precedes the clinical onset of the disease^50^, yet how it is connected to the pathogenesis of the disease remains to be established. Likewise, AD pathology includes mitochondrial dysfunction, yet how mitochondrial dysfunction arises and how it is mechanistically connected to AD-risk genes requires further studies^50^. Amyloid β, the major peptide accumulating in AD brains of both sporadic and hereditary forms of AD, has recently been shown to increase neuronal excitation due to inhibition of glutamate reuptake^51^. This prolonged neuronal signalling inevitably leads to increased Ca^2+^ influx and depletion of ATP, which in turn hyperactivate AMPK in AD^52,53^. This heightened AMPK activity directly increases mitochondrial fission and general autophagy^52^, but has also been reported to reduce mitophagy^53^. Whether amyloid β-induced neuronal hyperactivity and AMPK activation also impacts SYNJ2BP phosphorylation and *Pink1* mRNA localization remains to be determined. Also, other AD-associated genes impact mitochondrial function. This is the case for the AD risk factor ApoE4, which for example induces decreased mitochondrial respiration in the cortex of humanized aged male ApoE4 but not ApoE3 mice^54^. Our results now suggest that, in vitro, mitochondrial dysfunction in neurons may arise as a direct consequence of insulin resistance caused by the binding of ApoE4 to the IR^19^. Addition of ApoE4 successfully prevented the inhibition of AMPK upon insulin treatment, unlike addition of its homologue ApoE3 (Extended Data Fig. 7a). Accordingly, ApoE4 addition prevented the untethering of *Pink1* mRNA (Fig. 7a–c) and effective labelling of damaged mitochondria with p-ubiquitin in response to insulin (Fig. 7f,g), skewing the balance toward reduced PINK1 activity. It is tempting to speculate that this may contribute to the mitochondrial defects observed in ApoE4 mice^54^; however, altered insulin signalling in vivo over long time spans will also induce transcriptional regulation of mitochondrial gene expression as well as mTOR mediated effects^16^, hence, the exact contribution of altered mitochondrial quality control will have to be determined experimentally.

In general, our identification of a direct connection between insulin signalling and neuronal PINK1 biogenesis integrates into the ever-growing body of literature that connects failures in mitochondrial quality control and decline in insulin-related signalling pathways with general ageing and age-related diseases^50^. AMPK activation extends the life span in multiple organisms, including mice^55^, yet how AMPK activity can be used to prevent or treat neurodegenerative diseases remains controversial^56^. Our results reveal that both metabolic states (insulin signalling off/AMPK on versus insulin signalling on/AMPK off) are important for proper quality control of neuronal mitochondria by the PINK1/Parkin-dependent pathway, suggesting that AMPK dysregulation will have detrimental effects on mitochondrial quality control. While AMPK activation allows the efficient transport of *Pink1* mRNA to distal neurites, the second state then provides the cue to activate PINK1 and improve mitochondrial quality. Loss of either of these states, as seen pathologically during insulin resistance, will inevitably reduce the neuronal capacity to eliminate damaged organelles and contribute to the oxidative damage of the cell.

## Methods

### Mice

C57BL/6-N mice were housed in the animal facility of the Max Planck Institute for Biological Intelligence, Martinsried at 22 ± 1.5 °C and 55 ± 5% humidity with a 14:10-h light–dark cycle. All mouse procedures were performed according to the regulation of the Government of Upper Bavaria and approved by the Max Planck Institute for Biological Intelligence.

### Cell culture

#### Primary mouse neurons and transfection

Primary mouse neurons were prepared as described^3^. In brief, at E16.5 the pregnant mice were killed using CO_2_ and embryos of both sexes were extracted. Cortices and hippocampi were dissected in ice-cold dissociation medium (Ca^2+^-free Hank’s balanced salt solution with 100 mM MgCl_2_, 10 mM kynurenic acid and 100 mM HEPES). After enzymatic dissociation using papain/l-cysteine (Sigma-Aldrich), trypsin inhibitor (Abnova) was added to the tissue and titrated by pipetting. Neurons were resuspended in Neurobasal medium supplemented with B27 (Thermo Fisher Scientific), penicillin/streptomycin and l-glutamine (NB + B27 + PSG) and plated on 20 μg ml^−1^ poly-l-lysine (Sigma-Aldrich) and 3.5 μg ml^−1^ laminin (Thermo Fisher Scientific) coated glass-bottom plates (CellVis) or acid-washed glass coverslips (1.5 mm, Marienfeld). The medium was replaced 1:1 every 5 days with NB + B27 + PSG. Hippocampal neurons were seeded in 24-well glass-bottom plates (CellVis) at a density of 100 × 10^3^ or on glass coverslips (1.5 mm, Marienfeld) at a density of 50 × 10^3^ (immunocytochemistry) or 100 × 10^3^ (PLA). Transfection was conducted at day in vitro (DIV) 5–7 for 20 min using lipofectamine 2000 transfection reagent (Thermo Fisher Scientific) in NB + PSG. All cells were fixed or imaged after 2 days unless otherwise specified. Cortical neurons were seeded in six-well plates (Greiner) at a density of 2 × 10^6^.

#### iPS cell-derived cortical neurons

Human iPS cells (cell line: HPSI0314i-hoik_1) were obtained from the Wellcome Trust Sanger Institute HipSci Repository. iPS cells were maintained in StemFlex medium (Thermo Fisher Scientific) on Matrigel (Corning)-coated plates. The cells were passaged at 80% confluency.

iPS cells were differentiated into cortical neurons by overexpressing neurogenin-2 (NGN-2) as described^57^. In brief, iPS cells were dissociated into single cells using Accutase (Thermo Fisher Scientific) and plated at a density of 2.5 × 10^6^ per 10-cm dish (Falcon) in StemFlex supplemented with 10 µM Y-27632 (Tocris) (day −2). On day −1, iPS cells were transduced with lentivirus-packaged pLV-TetO-hNGN2-eGFP-puro and FudeltaGW-rtTA plasmids. On day 0, the medium was replaced with N2/DMEM/F12/NEAA (N2 medium; Thermo Fisher Scientific) containing 10 ng ml^−1^ BDNF (PeproTech), 10 ng ml^−1^ NT-3 (PeproTech) and 0.2 µg ml^−1^ laminin (Thermo Fisher Scientific), to which 2 µg ml^−1^ doxycycline (Takara) was added. On day 1, 1 µg ml^−1^ puromycin (Enzo Life Sciences) was added to the N2 medium. On days 2, 3 and 5, the medium was replaced with B27/Neurobasal-A/Glutamax (B27 medium; Thermo Fisher Scientific) containing 10 ng ml^−1^ BDNF, 10 ng ml^−1^ NT-3, 0.2 µg ml^−1^ laminin, 2 µg ml^−1^ doxycycline and 2 µM Ara-C (Sigma-Aldrich). On day 7, cells were cultured in NGN-2 glial conditioned Südhof neuronal growth medium containing 10 ng ml^−1^ BDNF, 10 ng ml^−1^ NT-3 and 0.2 µg ml^−1^ laminin. On day 8, iPS cell-derived cortical neurons were dissociated using TrypLE Express (Thermo Fisher Scientific) for 5 min at 37 °C and re-plated in 20 μg ml^−1^ poly-l-lysine (Sigma- Aldrich) and 3.5 μg ml^−1^ laminin-coated (Thermo Fisher Scientific) six-well plates (Greiner) at a density of 2 × 10^6^ cells. The medium was replaced 1:1 every other day with fresh conditioned Südhof neuronal growth medium. All treatments were carried out on DIV14.

#### Human embryonic kidney 293T cells

HEK293T cells were purchased from ATCC (CRL-11268G-1) and cultured in Dulbecco’s modified Eagle medium (Thermo Fisher Scientific) containing 10% fetal bovine serum. The cells were passaged at 80% confluency and seeded at a density of 0.5 × 10^6^ cells in six-well plates (Greiner).

### Treatments

Cells were treated before collection or imaging with AICAR (1 mM, 2 h, Abcam), CC (20 µM, 2 h, Abcam), AKT inhibitor VIII (10 µM, 2 h, TCI), GSK1904529A (1 µM, 2 h, Abcam), Wortmannin (1 µM, 2 h, EMD Millipore), CCCP (20 µM, 2 h, Sigma-Aldrich, insulin (500 nM, 1 h, Sigma-Aldrich), Torin-2 (10 nM, 30 min, Sigma-Alrich), AA (5 nM or 20 µM, 45 min, Alfa Aesar), ApoE3 or E4 (50 nM, overnight, PeproTech). The compounds were either dissolved in water (AICAR, CC, insulin, ApoE3 and E4), in dimethylsulfoxide (DMSO; AKT inhibitor VIII, GSK1904529A; Wortmannin, CCCP and Torin-2) or ethanol (AA). For compounds dissolved in DMSO or ethanol, control samples were treated with the same amount of the respective solvent. For insulin starvation, neurons were cultured for 2 h or overnight in NB + B27 + PSG lacking insulin.

### DNA constructs

Mito-mRaspberry-7, mito-meGFP, mito-BFP, EBFP2-C1 (cell fill), iRFP-C1 (cell fill), YFP-Parkin, pPBbsr2-4031NES (AMPK FRET biosensor), snap-OMP25 (SNAPmito), iRFP-Rab7, pLAMP1–mCherry, pLV-TetO-hNGN2-eGFP-puro and FudeltaGW-rtTA were acquired from Addgene (55931, 172481, 49151, 54665, 54786, 23955, 105241, 69599, 51613, 45147, 79823 and 19780, respectively). Plasmids encoding shRNA against SYNJ2BP, AMPK α1 and α2, and non-targeting (TRCN0000139049, TRCN0000024000, TRCN0000024046 and TR30021, respectively) were purchased in pLKO from Sigma-Aldrich. PINK1-kinase dead-MS2-PP7, Cox4i-MS2-PP7, Atp5f1b-MS2-PP7, split Venus, shRNA-resistant myc-tagged SYNJ2BP WT, SYNJ2mito WT and VQL/AAA plasmids have previously been described^3^. SYNJ2BP mutations S21A and S21E were introduced by site-directed mutagenesis. PINK1–GFP was generated in pHAGE by restriction-free cloning. The lentiviral packaging plasmids pMDLg/pRRE, pRSV-Rev and pMD2.G were acquired from Addgene (12251, 12253 and 12259, respectively). For purification of SYNJ2BP from *E.* *coli*, the cytosolic domain of rat SYNJ2BP WT (amino acids 1–110) was inserted into the bacterial expression vector pET19b and C-terminally tagged with 6xHis-tag.

### Protein purification of recombinant SYNJ2BP

Rosetta *E.* *coli* bacteria were transformed with pET19b-SYNJ2BP-6xHis WT or S21A. Bacteria were inoculated in 15 ml ZY auto-induction medium overnight, before fermenter inoculation at OD 0.04. After 24 h, bacteria were centrifuged at 5,000*g* for 10 min at 4 °C to yield a pellet of 50 g. Half the pellet was resuspended in 100 ml lysis buffer, consisting of His-Binding buffer (50 mM Na_3_PO_4_/Na_2_HPO_4_ pH 8, 500 mM NaCl, 10 mM imidazole, 10% glycerol) supplemented with 1 mM AEBSF-HCl, 2 µg ml^−1^ Aprotinin, 1 µg ml^−1^ Leupeptin, 1 µg ml^−1^ Pepstatin, 2.4 U ml^−1^ Benzonase Corefa and 2 mM MgCl_2_. Bacteria were lysed using an Avestin system and the lysate was centrifuged at 62,000*g* for 30 min at 4 °C.

The his-tagged proteins were purified via NiNTA-affinity chromatography using a linear gradient from 4–100% elution buffer (50 mM Na_3_PO_4_/Na_2_HPO_4_ pH 8, 500 mM NaCl, 250 mM imidazole and 10% glycerol) over ten column volumes and collected in 1-ml fractions. Pooled fractions were further purified by subsequent size-exclusion chromatography using a HiLoad 16/60 Superdex 75 column in SE/storage buffer (20 mM Tris pH 7.2, 30 mM NaCl and 10% glycerol). The samples were concentrated to 1 mg ml^−1^ in two batches using Amicon Ultra 15 columns and frozen at −80 °C.

### In vitro phosphorylation assays

Before phosphorylation, recombinant SYNJ2BP (WT or S21A, 0.5 µg) was dephosphorylated using 1 µl of CIP (NEB) in 9 µl 1× CutSmart buffer. The reactions were incubated at 37 °C for 1 h and stopped by 1× PhosStop (Roche). In vitro phosphorylation of dephosphorylated recombinant SYNJ2BP (0.5 µg in 10 µl) was performed in kinase reaction buffer (8 mM MOPS/NaOH, pH 7 and 200 µM EDTA) with 500 µM ATP (Serva), 200 µM AMP (Serva) and active recombinant AMPK (16 ng) (Sigma-Aldrich, 14-840) in a total volume of 30 µl. Alternatively, neuronal lysates were prepared from cells by Dounce homogenization in lysis buffer (20 mM Tris/HCl, pH 7.2, 30 mM NaCl, 10 mM MgCl_2_, 10% glycerol, 1 mM EDTA, 200 µM PMSF, protease inhibitor cocktail (Roche) and PhosStop (Roche)) and cleared by centrifugation at 2,300*g* for 1 min at 4 °C. The supernatant was collected and immediately used for in vitro phosphorylation assays or snap-frozen. Then, 0.5 µg SYNJ2BP was mixed with 25 µl cytosolic extract supplemented with 500 µM ATP in a final volume of 40 µl. The samples were incubated at 30 °C for 2 h (shaking). The reactions were stopped by addition of Laemmli sample buffer at 95 °C for 5 min.

### Phos-tag SDS–PAGE

In vitro phosphorylation assay samples of SYNJ2BP were analysed using Phos-tag SDS–PAGE. Standard discontinuous 20% polyacrylamide gels were prepared with 50 µM Phos binding reagent acrylamide (PBR-A and APExBIO) and 100 µM Zn(NO_3_)_2_. Before blotting on PVDF, the gel was incubated for 30 min in standard transfer buffer containing 1 mM EDTA and for 10 min in transfer buffer without EDTA. Membranes were decorated with anti-SYNJ2BP rabbit antibody (1:500 dilution; Proteintech, 15666-1-AP).

### Western blot analysis of protein levels

If indicated, cortical neurons were lentivirally transduced with myc-tagged SYNJ2BP WT, S21A and S21E, control shRNA, AMPKα1 or α2 shRNA on DIV1 and collected on DIV5–6. iPS cell-derived neurons and HEK293T cells were collected on DIV14 and 2 days after plating, respectively. Cells were lysed in lysis buffer (25 mM Tris/HCl, pH 7.4, 150 mM NaCl, 1% NP-40 and 1 mM EDTA supplemented with 200 µM PMSF and protease inhibitor cocktail (Roche)) and centrifuged at 9,300*g* for 1 min. The samples were analysed by gel electrophoresis on a 7.5–15% SDS–PAGE gel and immunoblotting using anti-SYNJ2BP rabbit (1:500 dilution; Proteintech, 15666-1-AP), anti-SYNJ2 rabbit (1:500 dilution; Proteintech, 13893-1-AP) anti-PINK1 rabbit (1:500 dilution; Novus Biologicals, BC100-494), anti-β-actin mouse (AC-74) (1:500 dilution; Sigma-Aldrich, A5316), anti-phospho-ubiquitin (E2J6T) rabbit (1:500 dilution; CST, 62802), anti-AMPKα1 rabbit (Y365) (1:500 dilution; Abcam, ab32047), anti-AMPKα2 mouse (A6A10) (1:500 dilution; Invitrogen, MA5-42560) and anti-βIII tubulin (2G10) mouse (1:2,000 dilution; Invitrogen, MA1-118) antibodies.

### Lentivirus production

Lentiviral particles were produced in HEK293T cells. On day −1, HEK293T cells were seeded in collagen-coated (Sigma-Aldrich) 10-cm dishes (Falcon) at a density of 6 × 10^6^ cells per dish. On day 0, cells were transfected with 5 µg packaging plasmid mix (pMDLg/pRRE, pRSV-Rev and pMD2.G; ratio 4:1:1) and 5 µg transfer plasmid (pLV-TetO-hNGN2-eGFP-puro, FudeltaGW-rtTA, myc-tagged SYNJ2BP WT, S21A or S21E) using TransIT-Lenti reagent (Mirus Bio). On day 2, the medium containing lentiviral particles was mixed with lentivirus precipitation solution (Alstem; ratio 4:1) and incubated at 4 °C overnight. On day 3, the lentiviral particles were collected by centrifugation at 1,500*g* for 30 min, resuspended in 1 ml ice-cold PBS/dish and stored at −80 °C.

### Co-immunoprecipitation

On DIV1, neurons were transduced with lentivirus-packaged myc-tagged SYNJ2BP WT or S21A plasmid. On DIV6, cells were cultured in insulin-free NB + B27 + PSG for 2 h, lysed in lysis buffer as above and centrifuged at 9,300*g* for 1 min. The supernatant was incubated either with or without CIP at 37 °C for 1 h and then with 2 µg anti-SYNJ2BP antibody (Proteintech, 15666-1-AP) per ml lysate for 30 min at 4 °C. Protein A Sepharose beads (10 mg per sample) were blocked with 1% BSA and added to the supernatant with the antibody. After 60 min incubation at 4 °C, beads were collected in columns and washed 5× (20 mM Tris/HCl, pH 8, 140 mM NaCl, 5 mM MgCl_2_, 0.5 mM dithiothreitol, 0.1% Triton-X and 200 µM phenylmethylsulfonyl fluoride). Proteins were eluted by addition of Laemmli sample buffer and boiled at 95 °C for 2 min. The samples were analysed by gel electrophoresis on a 7.5–15% gradient SDS–PAGE gel and immunoblotted as above.

### Phospho-MS

#### Sample preparation

On DIV1, neurons were transduced with lentivirus-packaged myc-tagged SYNJ2BP WT plasmid. On DIV6, the cells were cultured in insulin-free NB + B27 + PSG for 2 h and treated with or without CC and insulin, respectively. The neurons were collected in ice-cold PBS and centrifuged for 1 min at 9,300*g*. The pellets were snap-frozen and stored at −80 °C. The cell pellets (~10 × 10^6^ mouse cortical neurons) were incubated with 400 µl preheated sodium deoxycholate buffer (1% sodium deoxycholate (Sigma-Aldrich), 40 mM 2-chloroacetamide (Sigma-Aldrich) and 10 mM tris(2-carboxyethyl)phosphine (Thermo Fisher Scientific) in 100 mM Tris, pH 8.0). Afterwards, the samples were boiled for 5 min at 95 °C and ultrasonicated for 10 min with 10 × 30 s at high intensity and a 30-s pause between each cycle (Bioruptor Plus sonication system, Diagenode). Incubation and ultrasonication was repeated. The samples were diluted 1:1 with MS grade water (VWR). Proteins were digested with 2 µg Lys-C (Wako) for 4 h and overnight at 37 °C with 4 µg trypsin (Promega). The solution of peptides was acidified with trifluoroacetic acid (TFA; Merck) to a final concentration of 1%, followed by desalting via Sep-Pak C18 5cc vacuum cartridges (Waters).

#### Phospho-peptide enrichment

Phospho-peptide enrichment was performed in an automated fashion on an Assay MAP Bravo Platform (Agilent Technologies) as described^58^. In brief, the dried peptides were resuspended in 220 µl equilibration buffer (80% acetonitrile and 0.1% TFA). The peptides were loaded on a Assay MAP Fe(III)-NTA cartridge (Agilent Technology), washed with 200 µl priming buffer (100% acetonitrile and 0.1% TFA) and equilibrated with 250 µl equilibration buffer. After sample loading, the cartridge was washed with 250 µl equilibration buffer, eluted with 35 µl elution buffer (10% NH_3_ in H_2_O) and acidified with 10% formic acid (FA). Samples were concentrated in a SpeedVac for 45 min at 37 °C.

#### LC–MS/MS data acquisition

Liquid chromatography–tandem MS (LC–MS/MS) was performed on an Easy-nLC 1200 (Thermo Fisher Scientific) nanoflow system connected to an Orbitrap Exploris 480 mass spectrometer or a Q Exactive HF mass spectrometer (Thermo Fisher Scientific). Chromatographic separation was achieved on a 30-cm column (inner diameter, 75 μm; packed in-house with ReproSil-Pur C18-AQ 1.9-μm beads, Dr. Maisch). Peptides were injected onto the column in buffer A (0.1% FA) while heating the column at 60 °C. Peptides were eluted at a flow rate of 250 nl min^−1^ and a gradient of 2% of buffer B (80% acetonitrile and 0.1% FA) to 30% of buffer B over 100 min (Orbitrap Exploris 480) or 120 min (Q Exactive HF) followed by a ramp to 60% over 10 min then 95% over the next 5 min and maintained at 95% for another 5 min.

The mass spectrometer Exploris 480 was operated in a data-dependent mode with survey scans from 300–1,650 m/z (resolution of 60,000 at m/z of 200) and the top 15 precursors were selected and fragmented using higher energy collisional dissociation (HCD with a normalized collision energy of 30). For the measurement of enriched phospho-peptides, the MS2 spectra were recorded at 30,000 (m/z of 200) and for the measurement of the total proteomes, the MS2 spectra were recorded at 15,000 (m/z of 200). Normalized AGC targets for MS1 and MS2 scans were set to 300% and 100%, respectively, within a maximum injection time of 25 ms for the MS1 scan. Similar parameters were applied to the Q Exactive HF measurements.

#### Data analysis

Raw data were processed using the MaxQuant computational platform (v.2.0.1.0)^59^ with standard settings applied. In brief, the peak list was searched against the Uniprot mouse proteome data base and the sequence of SYNJ2BP (rat) with an allowed precursor mass deviation of 4.5 ppm and an allowed fragment mass deviation of 20 ppm. Cysteine carbamidomethylation was set as static modification. Methionine oxidation, N-terminal acetylation, deamidation on asparagine and glutamine and phosphorylation on serine, threonine and tyrosine were set as variable modifications. The match-between runs option was enabled. The label-free quantification algorithm (MaxQuant) was used for quantification of proteins and the iBAQ algorithm (MaxQuant) was used for calculation of approximate abundances for the identified proteins.

### RNA isolation and RT–qPCR

Before RNA isolation, respective treatments of the neurons were performed: 20 µM CC (Abcam) for 2 h, 50 nM ApoE3 (PeproTech) or 50 nM ApoE4 (PeproTech) overnight. On DIV6, RNA was isolated using the QIAGEN RNeasy Mini kit. Complementary DNA was generated using the qScript cDNA SuperMix (Quantabio) followed by quantitative PCR with reverse transcription (RT–qPCR) using the PerfeCTa SYBR Green FastMix (Quantabio) in a Mic (magnetic induction cycler) PCR machine (Bio Molecular Systems). The following primers were used (5′→ 3′): Pink1 forward: AATGAGCCAGGAGCTGGTC; Pink1 reverse: GTACTGGCGCAGGGTACAG; β-actin forward: ACACTGTGCCCATCTACG; β-actin reverse: GCTGTGGTGGTGAAGCTGTAG. *Pink1* transcript levels upon CC treatment were determined relative to β*-*actin and control treatment using the comparative Ct method (formula: 2^-ΔΔCt^).

### Live-cell imaging

Live-cell mRNA imaging of *Pink1*, *Cox4i* and *Atp5f1b* in primary mouse hippocampal neurons was performed as described^3^. In brief, the optimal ratio between the Pink1, Cox4i or Atp5f1b and the split-Venus construct was empirically determined to be 4:1. Control, SYNJ2BP or AMPK shRNAs were expressed for 4 days to ensure effective knockdown and expression of YFP-Parkin or PINK1–GFP was allowed for 2 days. On DIV9, imaging was performed in Hibernate E medium (BrainBits) at the Imaging Facility of the Max Planck Institute for Biological Intelligence, Martinsried, with an Eclipse Ti2 spinning-disk microscope equipped with a DS-Qi2 high-sensitivity monochrome camera using a ×60 NA 1.40 oil-immersion lens and NIS-Elements software (Nikon). For Parkin translocation, images of the same neurons were taken before and 30 min after addition of AA.

### Immunostainings

Neurons were fixed with 4% paraformaldehyde for 15 min, permeabilized with 0.3% Triton-X-100/PBS for 10 min and blocked for 1 h at room temperature (1% BSA/PBS). Neurons were incubated with primary antibodies diluted in blocking buffer at 4 °C for 1 h or overnight. The following primary antibodies were used: mouse anti-SYNJ2BP (1:50 dilution; Sigma-Aldrich, SAB1400613), rabbit anti-phospho-ubiquitin S65 (1:200 dilution; Millipore, ABS1513-I), rabbit anti-optineurin (1:500 dilution; Abcam, ab23666) and mouse anti-βIII tubulin (2G10) (1:1,000 dilution; Invitrogen, MA1-118). Neurons were incubated with secondary fluorescent antibodies (goat anti-mouse IgG (H + L) Alexa Fluor 568 (1:500 dilution; Invitrogen, A11004) and goat anti-rabbit IgG (H + L) Alexa Fluor Plus 647 (1:500 dilution; Invitrogen, A32733)) diluted in blocking buffer for 2 h at room temperature. The coverslips were mounted in Fluoromount G (Invitrogen) and imaged at an Eclipse Ti2 spinning-disk microscope (Nikon) using a ×60 NA 1.40 oil-immersion objective.

### Mitochondrial morphology and motility

On DIV9, imaging was performed in Hibernate E medium (BrainBits) supplemented with or without 500 nM insulin for 30 min or 20 µM CC for 2 h (Abcam) with an Eclipse Ti2 spinning-disk microscope using a ×60 NA 1.2 oil-immersion lens. For motility, images of the neurites were acquired every 500 ms for 3 min.

### PLA

A PLA was performed according to the manufacturer’s instructions (Sigma-Aldrich) and as described^3^. Neurons were fixed with 4% paraformaldehyde for 15 min and permeabilized with 0.3% Triton-X-100/PBS for 10 min followed by a 1 h incubation with Duolink blocking solution at 37 °C in a humidity chamber. Neurons were incubated with primary antibodies (mouse anti-SYNJ2BP, 1:50 dilution, Sigma-Aldrich, SAB1400613 and rabbit anti-SYNJ2, 1:50 dilution, Proteintech, 13893-1-AP) diluted in Duolink antibody diluent at 4 °C overnight. The neurons were washed and incubated with the Duolink PLA Probes (PLUS rabbit and MINUS mouse) at 37 °C for 1 h. This was followed by incubation with Duolink ligation solution at 37 °C for 30 min and amplification solution at 37 °C for 100 min. The coverslips were mounted in Fluoromount G (Thermo Fisher Scientific) and imaged with a Nikon Ti2 spinning-disk microscope using a ×60 NA 1.40 oil-immersion objective. The number of PLA puncta per soma was quantified and normalized to the cell body size.

### Fluorescence lifetime imaging

Imaging was performed with a LEICA (Wetzlar) SP8 FALCON confocal laser scanning microscope equipped with an HCX PL APO ×63 1.2 motCORR CS water-immersion objective using the LAS-X software (v.3.5.5). Neurons were excited with a pulsed diode laser (PicoQuant) at 440 nm and photon arrival times of a maximum of 1,000 photons per brightest pixel were detected between 470 and 512 nm. A fluorescence lifetime imaging phasor analysis approach was used to determine the fluorescence lifetime of the donor^60^.

### Quantification and statistical analysis

Data are expressed as mean ± s.e.m. Statistical analysis was performed in GraphPad Prism (v.9.1.0). Statistical significance was determined using either a Student’s *t*-test, Welch’s *t*-test or one-way analysis of variance (ANOVA) with Tukey’s or Dunnett’s multiple comparisons. All *P* values are given in the figures. No statistical methods were used to pre-determine sample sizes but our sample sizes are similar to those reported in previous publications^3^. Samples were randomly distributed into experimental conditions. Data distribution was assumed to be normal but this was not formally tested. Individual data points are shown and points with the same colour belong to the same biological replicate. Data collection and analysis were not performed blind to the conditions of the experiments (except in Fig. 6a,b).

Western blot images were acquired using the Invitrogen iBright FL1000 Imaging System (Thermo Fisher Scientific) and quantified in Fiji/ImageJ (v.2.14.0/1.54f; National Institutes of Health)^61^. Quantification of microscopy data was performed in Fiji/ImageJ. For neurites, images were straightened with a 20-pixel margin after maximum z-projection. A square-shaped region including the entire cell body was used for cell bodies and no maximum z-projection was performed. For *Pink1* mRNA imaging, the Manders’ colocalization coefficient of the Venus and the mitochondrial or late endosomal channel was analysed in z-stack images using the JaCoP plugin as described^3^. For the rotated Venus quantification, a 10 × 10-µm square was chosen within the cell body based on the mitochondrial signal. For mitochondrial morphology, the threshold for the image of the mitochondrial signal was chosen by eye. The image was smoothened twice, made binary and skeletonized. The skeleton was analysed and the average branch length per neuron was calculated. For motility of mitochondria in neurites, kymographs were generated using the Kymolyzer plugin^62^. To determine Parkin-positive mitochondria, plots of fluorescence intensity versus the position along neurites were generated using the plot profile function of Fiji/ImageJ. A mitochondrion was considered Parkin positive when the intensity of the respective YFP-Parkin peak was at least twice as high as the baseline intensity level.

### Reporting summary

Further information on research design is available in the Nature Portfolio Reporting Summary linked to this article.

### Supplementary information

Reporting Summary

Supplementary Video 1 Representative video of *Pink1* mRNA (green) visualized by the MS2/PP7-split-Venus method and Raspberry-labelled mitochondria (magenta) in a primary hippocampal neuron treated with 20 µM CC. Time-lapse imaging started 20 min after addition of CC, with images taken every 30 s. The white arrowhead marks a *Pink1* mRNA dot that dissociates from mitochondria. Scale bar, 10 µm.

Supplementary Video 2 Representative video of *Pink1* mRNA (green) visualized by the MS2/PP7-split-Venus method in a primary hippocampal neuron treated with 20 µM CC. Time-lapse imaging started 20 min after addition of CC, with images taken every 30 s. Scale bar, 10 µm.

Supplementary Video 3 Representative video of Raspberry-labelled mitochondria (magenta) in a primary hippocampal neuron treated with 20 µM CC. Time-lapse imaging started 20 min after addition of CC, with images taken every 30 s. Scale bar, 10 µm.

### Source data

Source Data Fig. 1 Statistical Source Data.

Source Data Fig. 2 Statistical Source Data.

Source Data Fig. 3 Statistical Source Data.

Source Data Fig. 3 Unprocessed western blots.

Source Data Fig. 4 Statistical Source Data.

Source Data Fig. 5 Statistical Source Data.

Source Data Fig. 6 Statistical Source Data.

Source Data Fig. 6 Unprocessed western blots.

Source Data Fig. 7 Statistical Source Data.

Source Data Extended Data Fig. 1 Statistical Source Data.

Source Data Extended Data Fig. 1 Unprocessed western blots.

Source Data Extended Data Fig. 2 Statistical Source Data.

Source Data Extended Data Fig. 2 Unprocessed western blots.

Source Data Extended Data Fig. 3 Statistical Source Data.

Source Data Extended Data Fig. 4 Statistical Source Data.

Source Data Extended Data Fig. 4 Unprocessed western blots.

Source Data Extended Data Fig. 5 Statistical Source Data.

Source Data Extended Data Fig. 5 Unprocessed western blots.

Source Data Extended Data Fig. 6 Statistical Source Data.

Source Data Extended Data Fig. 6 Unprocessed western blots.

Source Data Extended Data Fig. 7 Statistical Source Data.

Source Data Extended Data Fig. 8 Statistical Source Data.

Source Data Extended Data Fig. 8 Unprocessed western blots.

Source Data Extended Data Fig. 9 Statistical Source Data.

Source Data Extended Data Fig. 9 Unprocessed western blots.

Source Data Extended Data Fig. 10 Statistical Source Data.

## Data Availability

The datasets used during the study are available from the corresponding author on reasonable request. The MS data have been deposited to the ProteomeXchange Consortium (http://proteomecentral.proteomexchange.org) via the PRIDE partner repository with the dataset identifier PXD045621. Source data are provided with this paper.
