# Supplementary material for: Insulin signalling regulates Pink1 mRNA localization via modulation of AMPK activity to support PINK1 function in neurons
Source: Nat Metab. 2024 Mar 19;6(3):514–30. doi: 10.1038/s42255-024-01007-w (PMC10963278; doi:10.1038/s42255-024-01007-w)
Supplement: Supplementary file 1 — Reporting Summary [file 42255_2024_1007_MOESM1_ESM.pdf]

nature portfolio

Corresponding author(s): Angelika B. Harbauer

Last updated by author(s): Jan 17, 2024

Reporting Summary

Nature Portfolio wishes to improve the reproducibility of the work that we publish. This form provides structure for consistency and transparency in reporting. For further information on Nature Portfolio policies, see our Editorial Policies and the Editorial Policy Checklist.

Statistics

For all statistical analyses, confirm that the following items are present in the figure legend, table legend, main text, or Methods section.

n/a	Confirmed	
☐	☒	The exact sample size (*n*) for each experimental group/condition, given as a discrete number and unit of measurement
☐	☒	A statement on whether measurements were taken from distinct samples or whether the same sample was measured repeatedly
☐	☒	The statistical test(s) used AND whether they are one- or two-sided<br>*Only common tests should be described solely by name; describe more complex techniques in the Methods section.*
☒	☐	A description of all covariates tested
☐	☒	A description of any assumptions or corrections, such as tests of normality and adjustment for multiple comparisons
☐	☒	A full description of the statistical parameters including central tendency (e.g. means) or other basic estimates (e.g. regression coefficient) AND variation (e.g. standard deviation) or associated estimates of uncertainty (e.g. confidence intervals)
☐	☒	For null hypothesis testing, the test statistic (e.g. *F*, *t*, *r*) with confidence intervals, effect sizes, degrees of freedom and *P* value noted<br>*Give P values as exact values whenever suitable.*
☒	☐	For Bayesian analysis, information on the choice of priors and Markov chain Monte Carlo settings
☒	☐	For hierarchical and complex designs, identification of the appropriate level for tests and full reporting of outcomes
☒	☐	Estimates of effect sizes (e.g. Cohen's *d*, Pearson's *r*), indicating how they were calculated

Our web collection on statistics for biologists contains articles on many of the points above.

Software and code

Policy information about availability of computer code

Data collection	Bio Molecular Systems micPCR version 2.8.10 was used for RT-qPCR.<br>Nikon NIS-Elements version 5.21.03 was used to collect spinning disk confocal microscope images.<br>Leica Application Suite X (LAS-X) software version 3.5.5 was used to collect fluorescence lifetime microscopy (FLIM) images and confocal laser scanning microscope images.
Data analysis	MaxQuant computational platform version 2.0.1.0 (RRID:SCR_014485) was used for analysis of mass spectrometry data. (The iBAQ and LFQ algorithm are part of the MaxQuant computational platform.)<br>Fiji/ImageJ version 2.14.0/1.54f (National Institute of Health; RRID:SCR_002285) was used for analysis of microscopy images and Western blot densitometry.<br>Prism GraphPad Software version 9.1.0 (RRID:SCR_002798) was used for statistical analysis.

For manuscripts utilizing custom algorithms or software that are central to the research but not yet described in published literature, software must be made available to editors and reviewers. We strongly encourage code deposition in a community repository (e.g. GitHub). See the Nature Portfolio guidelines for submitting code & software for further information.

Data

Policy information about availability of data

All manuscripts must include a data availability statement. This statement should provide the following information, where applicable:

- Accession codes, unique identifiers, or web links for publicly available datasets
- A description of any restrictions on data availability
- For clinical datasets or third party data, please ensure that the statement adheres to our policy

The datasets used during the current study are available from the corresponding author on reasonable request. The mass spectrometry data have been deposited to the ProteomeXchange Consortium (http://proteomecentral.proteomexchange.org) via the PRIDE partner repository with the dataset identifier PXD045621.

Human research participants

Policy information about studies involving human research participants and Sex and Gender in Research.

Reporting on sex and gender	n/a
Population characteristics	n/a
Recruitment	n/a
Ethics oversight	n/a

Note that full information on the approval of the study protocol must also be provided in the manuscript.

Field-specific reporting

Please select the one below that is the best fit for your research. If you are not sure, read the appropriate sections before making your selection.

☒ Life sciences ☐ Behavioural & social sciences ☐ Ecological, evolutionary & environmental sciences

For a reference copy of the document with all sections, see nature.com/documents/nr-reporting-summary-flat.pdf

Life sciences study design

All studies must disclose on these points even when the disclosure is negative.

Sample size	The sample size was chosen based on common standards in the field and on our previous experience (PMID: 35216662), while taking into account the experimental effort to generate the respective data. No statistical method was used to pre-determine the sample size.
Data exclusions	No data were excluded from the analysis.
Replication	All experiments were repeated as described in the figure legends. All attempts at replication were successful.
Randomization	Cells were randomly allocated into experimental groups. Cells were plated in 6-well or 24-well plates. Each well was randomly assigned to a treatment group.
Blinding	As the experiments were performed and analyzed by the same investigator, the majority of the analyses was not blinded. For the analysis of the experiment in Fig. 6a,b, however, the investigator was blinded.

Reporting for specific materials, systems and methods

We require information from authors about some types of materials, experimental systems and methods used in many studies. Here, indicate whether each material, system or method listed is relevant to your study. If you are not sure if a list item applies to your research, read the appropriate section before selecting a response.

Materials & experimental systems

n/a	Involved in the study	
☐	☒	Antibodies
☐	☒	Eukaryotic cell lines
☒	☐	Palaeontology and archaeology
☐	☒	Animals and other organisms
☒	☐	Clinical data
☒	☐	Dual use research of concern

Methods

n/a	Involved in the study	
☒	☐	ChIP-seq
☒	☐	Flow cytometry
☒	☐	MRI-based neuroimaging

Antibodies

Antibodies used

Anti-SYNJ2BP rabbit Proteintech Cat# 15666-1-AP Lot#00068490
Anti-SYNJ2BP mouse Sigma-Aldrich Cat# SAB1400613 Lot# KA141
Anti-SYNJ2 rabbit Proteintech Cat# 13893-1-AP Lot# 00004764
Anti-bIII tubulin 2G10 mouse Invitrogen Cat# MA1-118 Lot# WK337755
Anti-b-actin AC-74 mouse Sigma-Aldrich Cat# A5316 Lot# 0000118499
Anti-PINK1 rabbit Novus biologicals Cat# BC100-494 Lot# 0-4
Anti-p-ubiquitin (S65) Millipore Cat# ABS1513-I Lot# 3845000
Anti-p-ubiquitin (S65) E2J6T rabbit Cell Signaling Cat# 62802 Lot# 3
Anti-AMPKa1 Y365 rabbit Abcam Cat# ab32047 Lot# GR3318895-25
Anti-AMPKa2 A6A10 mouse Invitrogen Cat# MA5-42560 Lot# YA3807886B
Anti-Optineurin rabbit Abcam Cat# ab23666 Lot# GR3336359-2
Goat anti-mouse IgG (H+L) Cross-Adsorbed Secondary Antibody Alexa Fluor 568 Invitrogen Cat# A11004 Lot# 2090670
Goat anti-rabbit IgG (H+L) Highly Cross-Adsorbed Secondary Antibody Alexa Fluor Plus 647 Invitrogen Cat# A32733 Lot# YI376194

Validation

All antibodies used are commercially available and have been validated by the manufacturer and/or other investigators as indicated on the websites:

Anti-SYNJ2BP rabbit - https://www.ptglab.com/products/SYNJ2BP-Antibody-15666-1-AP.htm. Further validation of this antibody using SYNJ2BP shRNA was done by our lab (PMID: 35216662).

Anti-SYNJ2BP mouse - https://www.sigmaaldrich.com/DE/de/product/sigma/sab1400613

Anti-SYNJ2 rabbit - https://www.ptglab.com/products/SYNJ2-Antibody-13893-1-AP.htm

Anti-bIII tubulin 2G10 mouse - https://www.thermofisher.com/antibody/product/MA1-118.html

Anti-b-actin AC-74 mouse - https://www.sigmaaldrich.com/DE/de/product/sigma/a5316

Anti-PINK1 rabbit - https://www.novusbio.com/products/pink1-antibody_bc100-494

Anti-p-ubiquitin (S65) rabbit Millipore - https://www.sigmaaldrich.com/DE/de/product/mm/abs1513i

Anti-p-ubiquitin (S65) E2J6T rabbit Cell Signaling - https://www.cellsignal.com/products/primary-antibodies/phospho-ubiquitin-ser65-e2j6t-rabbit-mab/62802

Anti-AMPKa1 Y365 rabbit - https://www.abcam.com/products/primary-antibodies/ampk-alpha-1-antibody-y365-ab32047.html

Anti-AMPKa2 A6A10 mouse - https://www.thermofisher.com/antibody/product/AMPK-alpha-2-Antibody-clone-A6A10-Monoclonal/MA5-42560

Anti-Optineurin rabbit - https://www.abcam.com/products/primary-antibodies/optineurin-antibody-ab23666.html

Goat anti-mouse IgG (H+L) Cross-Adsorbed Secondary Antibody Alexa Fluor 568 - https://www.thermofisher.com/antibody/product/Goat-anti-Mouse-IgG-H-L-Cross-Adsorbed-Secondary-Antibody-Polyclonal/A-11004

Goat anti-rabbit IgG (H+L) Highly Cross-Adsorbed Secondary Antibody Alexa Fluor 647 - https://www.thermofisher.com/antibody/product/Goat-anti-Rabbit-IgG-H-L-Highly-Cross-Adsorbed-Secondary-Antibody-Polyclonal/A32733

Eukaryotic cell lines

Policy information about cell lines and Sex and Gender in Research

Cell line source(s)

HEK293T cells were purchased from ATCC. Human induced pluripotent stem cells (iPSCs) were obtained from the Wellcome Trust Sanger Institute HipSci Repository.

Authentication

Cell lines were authenticated by morphology check under the microscope before each experiment.

Mycoplasma contamination	The HEK293T cells were tested negative for mycoplasma contamination by PCR. The human induced pluripotent stem cells were not tested for mycoplasma contamination.
Commonly misidentified lines (See ICLAC register)	No commonly misidentified cell lines were used.

Animals and other research organisms

Policy information about studies involving animals; ARRIVE guidelines recommended for reporting animal research, and Sex and Gender in Research

Laboratory animals	Species: Mouse; Strain: C57BL/6<br>E16.5 mouse embryos were used to obtain primary hippocampal and cortical cultures.
Wild animals	The study did not involve wild animals.
Reporting on sex	Brains from male and female embryos were pooled prior to dissection.
Field-collected samples	The study did not involve samples collected from the field.
Ethics oversight	All mouse procedures were performed according to the regulation of the Government of upper Bavaria (Germany).

Note that full information on the approval of the study protocol must also be provided in the manuscript.
