# Supplementary figures and images for: Insulin signalling regulates Pink1 mRNA localization via modulation of AMPK activity to support PINK1 function in neurons
Source: Nat Metab. 2024 Mar 19;6(3):514–30. doi: 10.1038/s42255-024-01007-w (PMC10963278; doi:10.1038/s42255-024-01007-w)

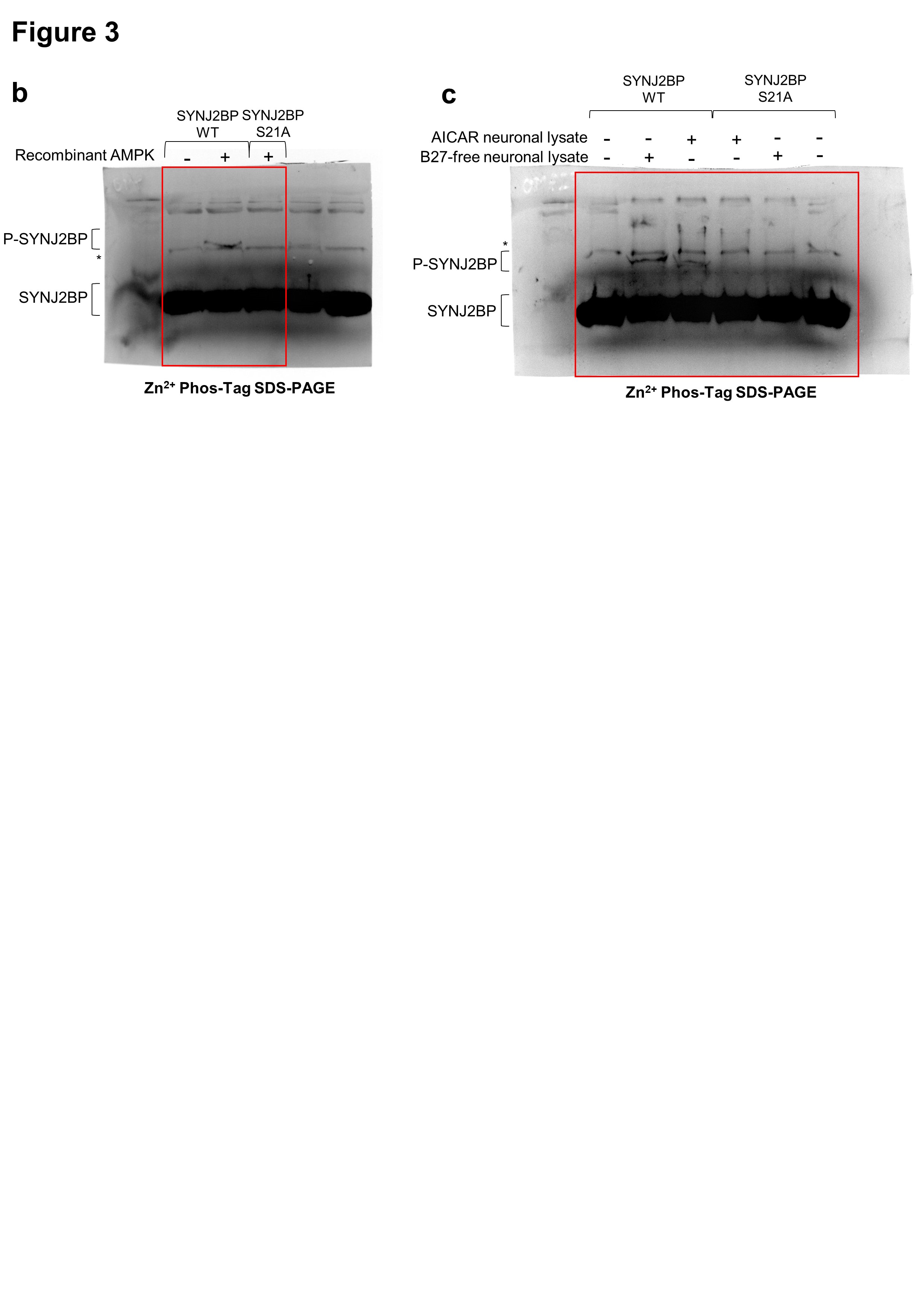

Supplement: Supplementary file 8 — Unprocessed western blots. [file 42255_2024_1007_MOESM8_ESM.jpg]

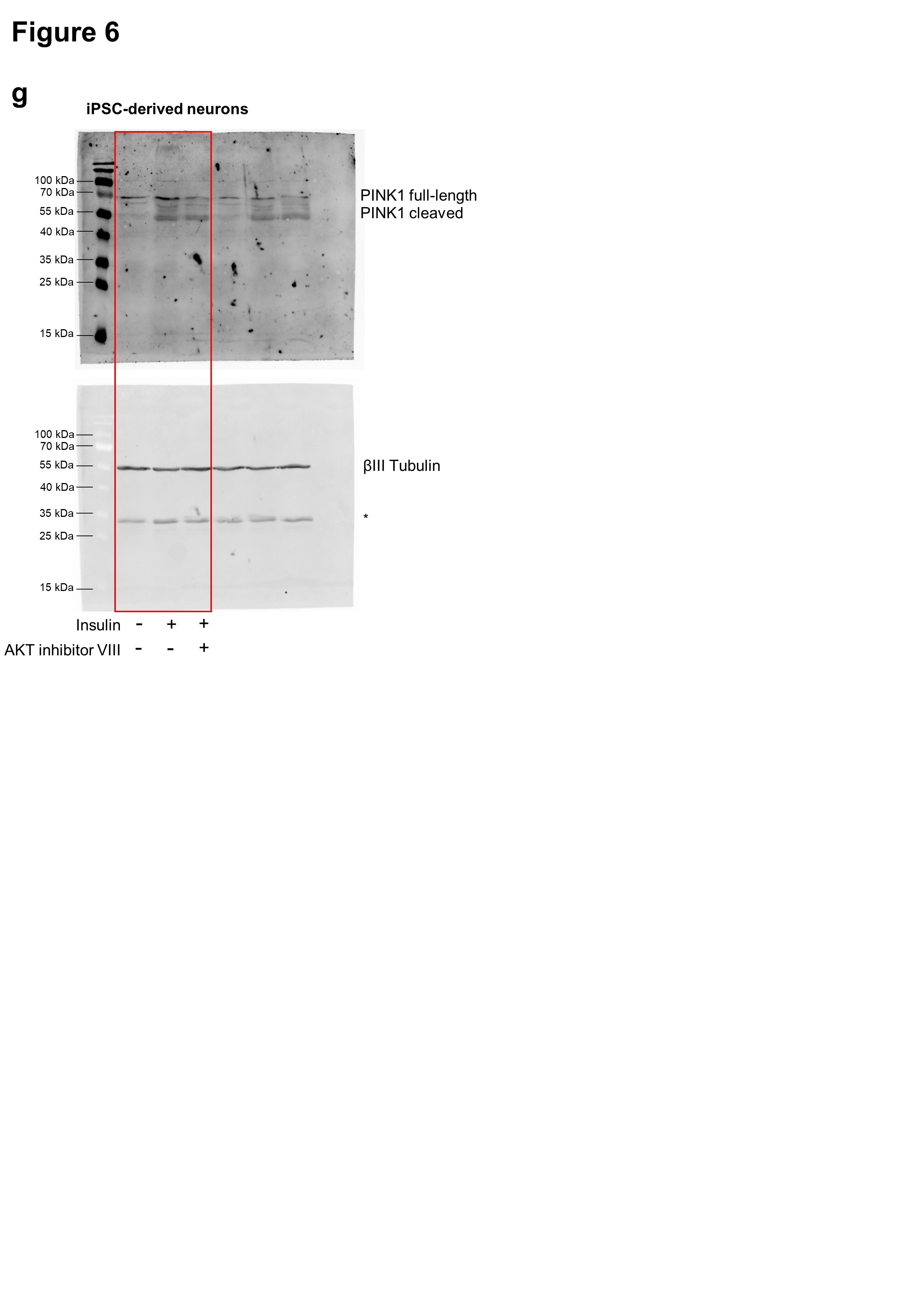

Supplement: Supplementary file 12 — Unprocessed western blots. [file 42255_2024_1007_MOESM12_ESM.jpg]

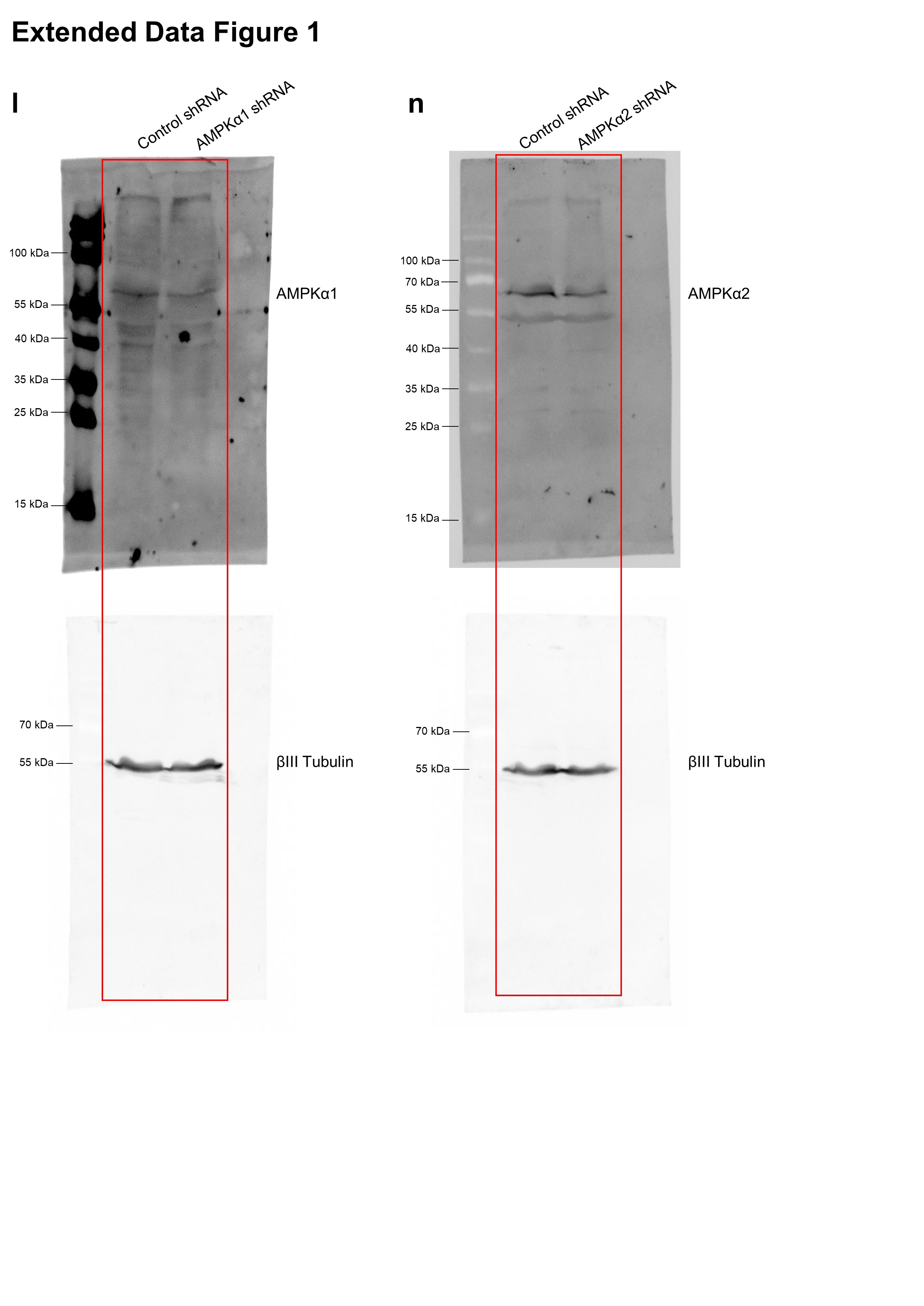

Supplement: Supplementary file 15 — Unprocessed western blots. [file 42255_2024_1007_MOESM15_ESM.jpg]

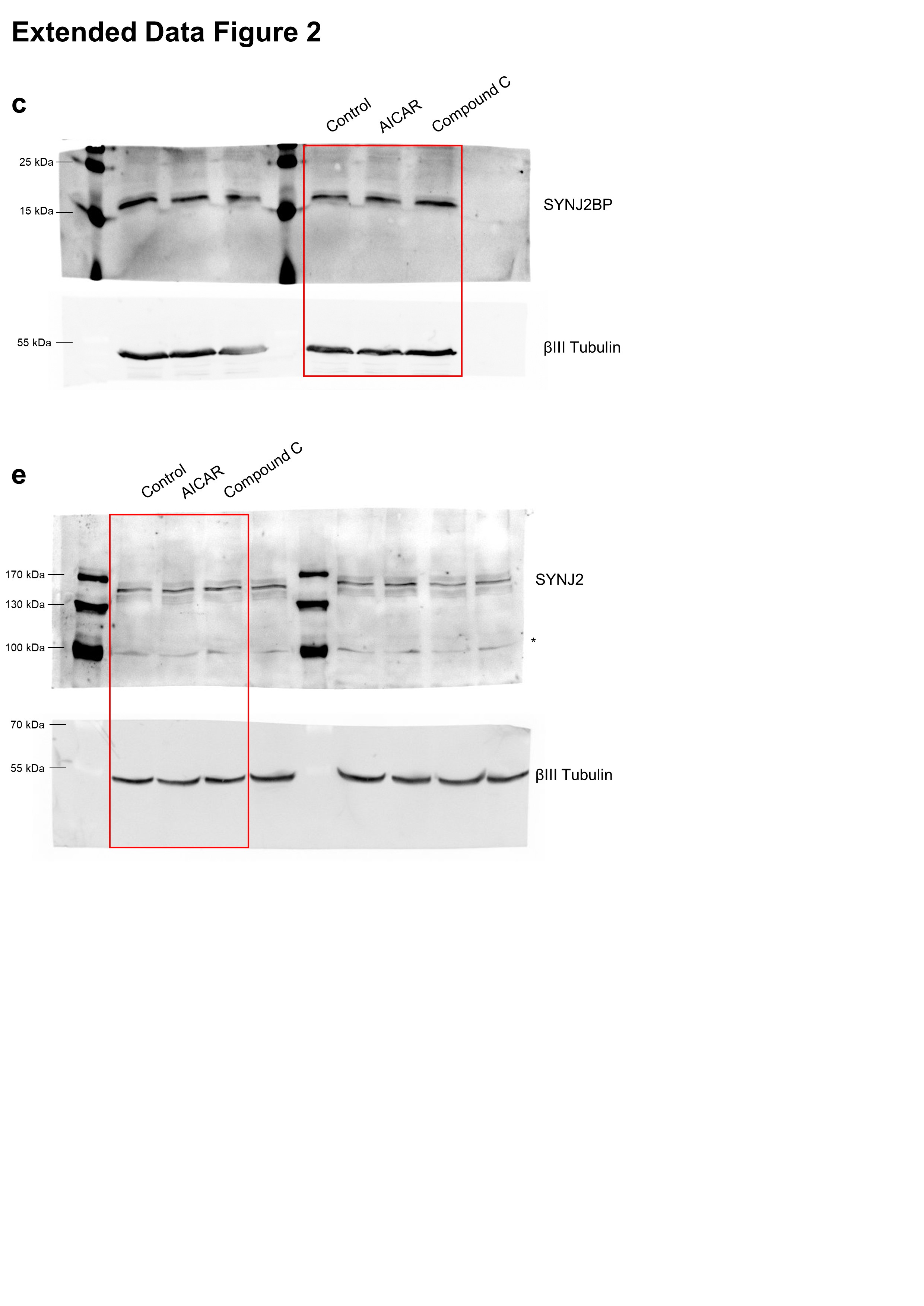

Supplement: Supplementary file 17 — Unprocessed western blots. [file 42255_2024_1007_MOESM17_ESM.jpg]

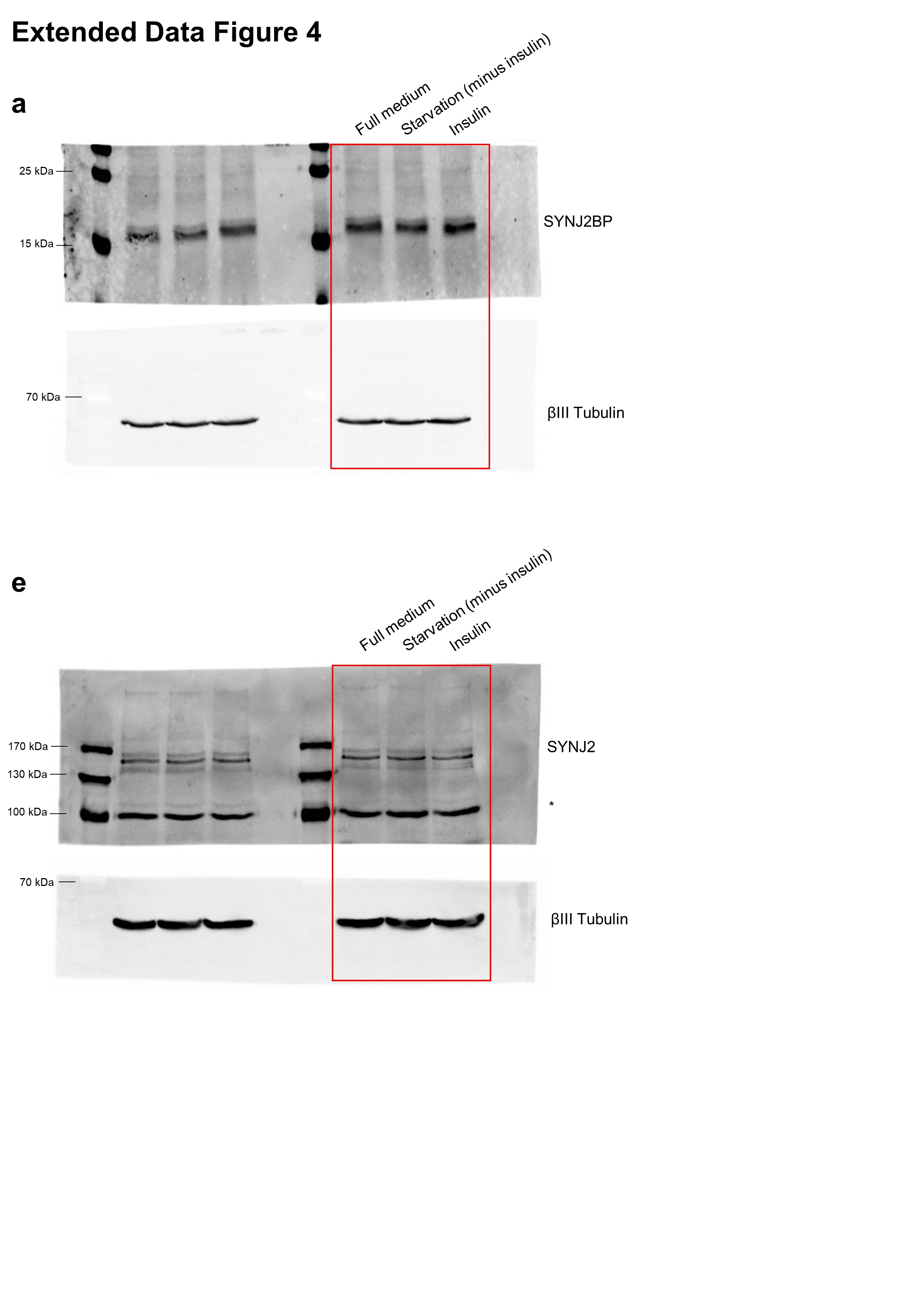

Supplement: Supplementary file 20 — Unprocessed western blots. [file 42255_2024_1007_MOESM20_ESM.jpg]

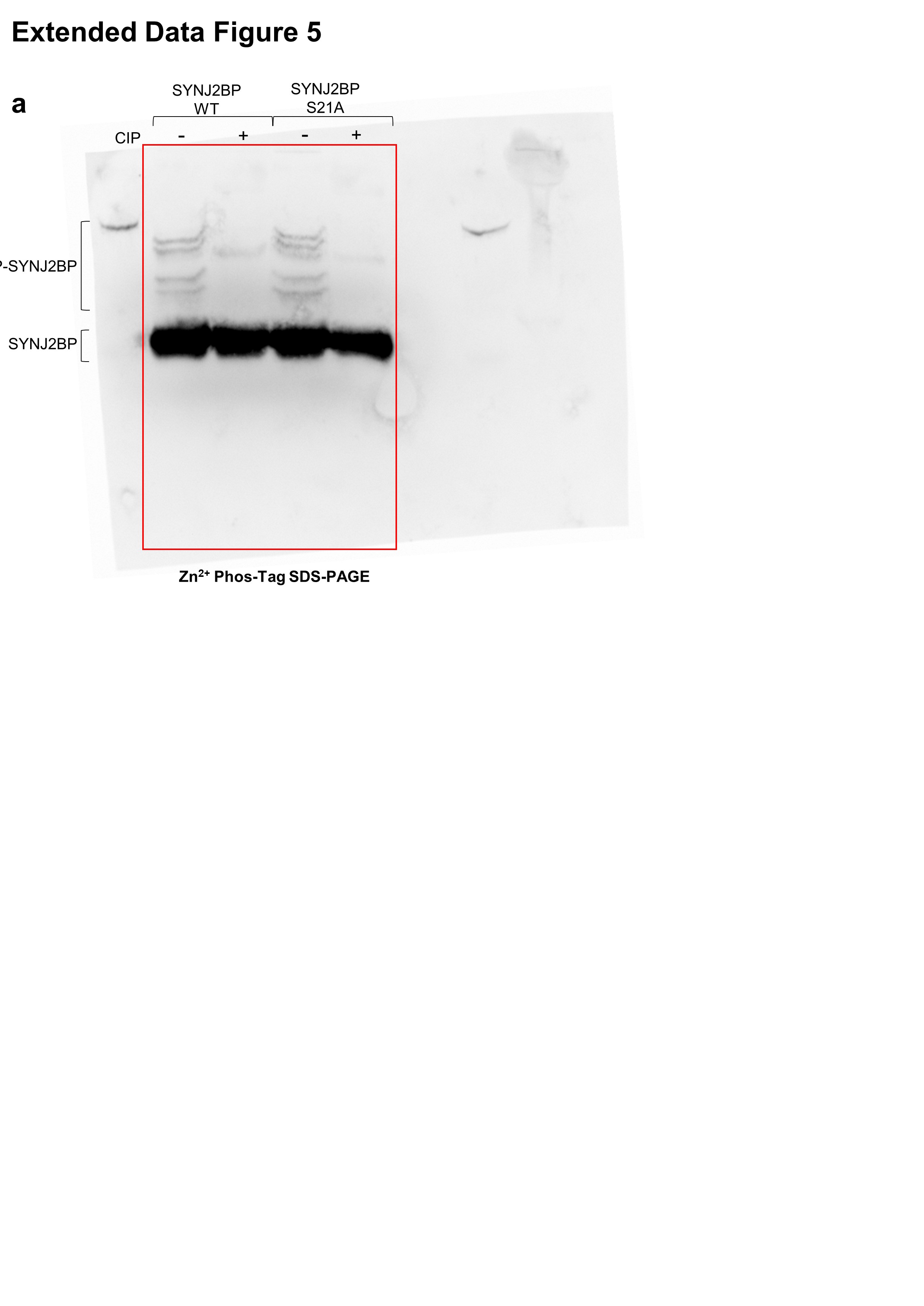

Supplement: Supplementary file 22 — Unprocessed western blots. [file 42255_2024_1007_MOESM22_ESM.jpg]

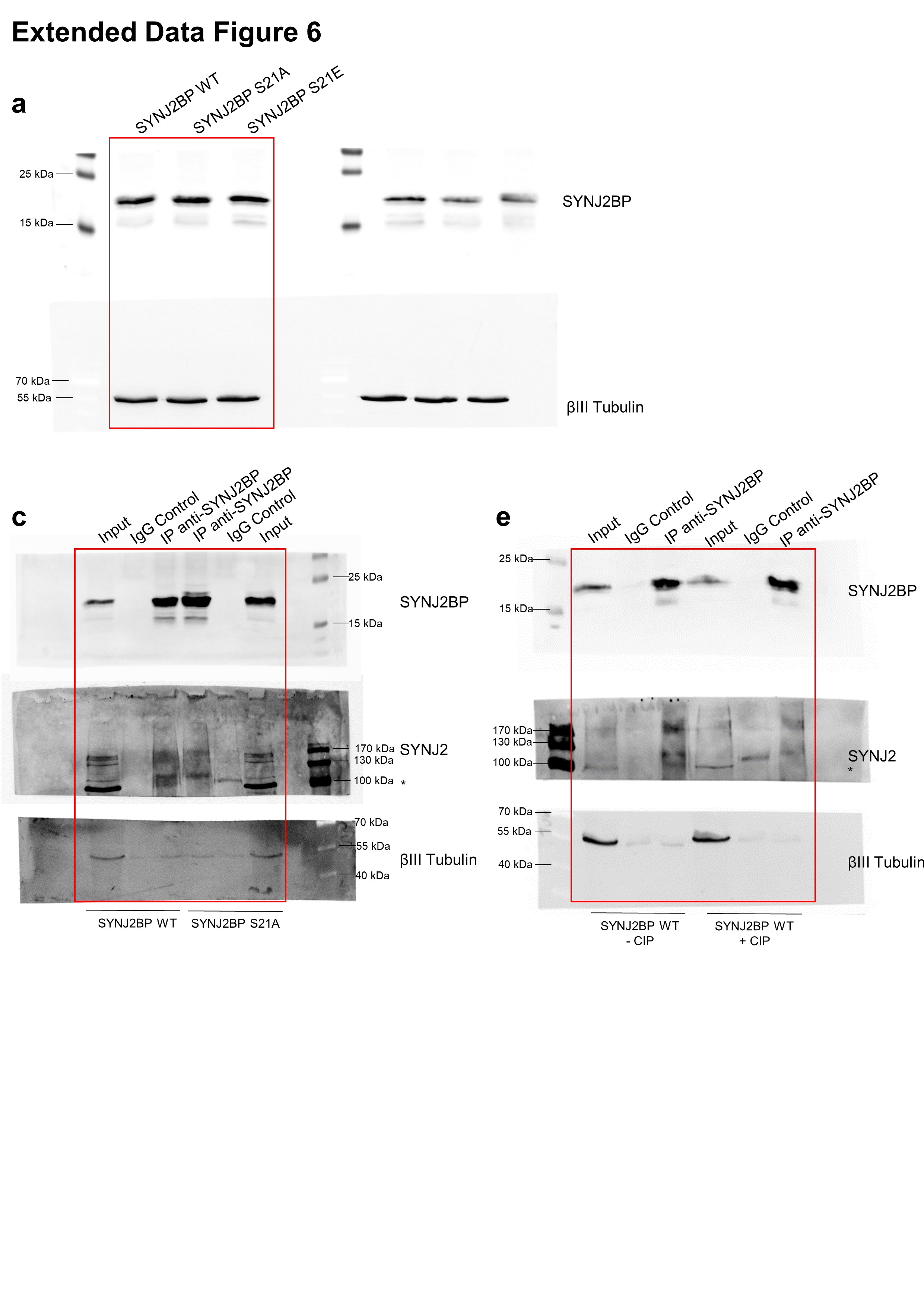

Supplement: Supplementary file 24 — Unprocessed western blots. [file 42255_2024_1007_MOESM24_ESM.jpg]

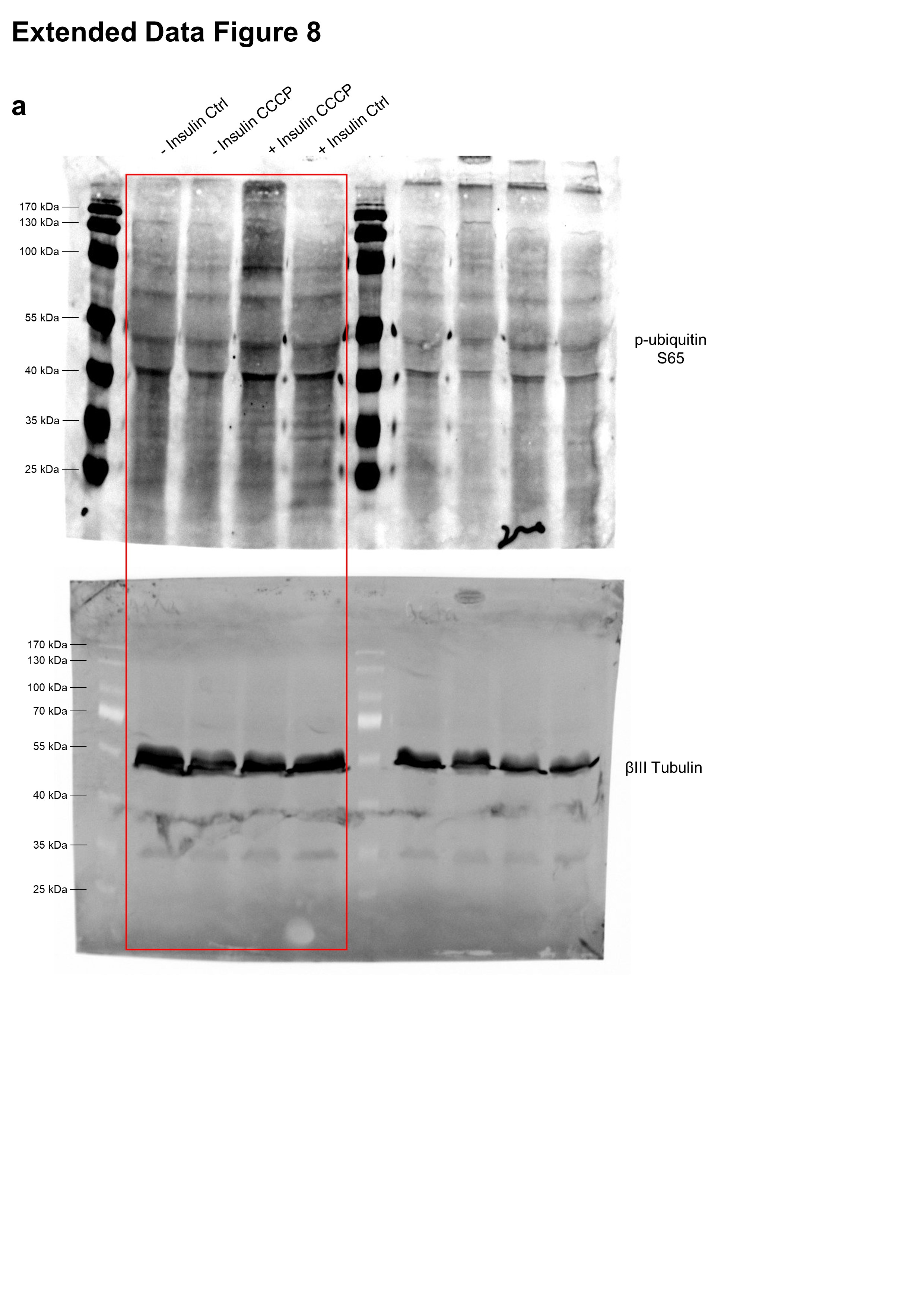

Supplement: Supplementary file 27 — Unprocessed western blots. [file 42255_2024_1007_MOESM27_ESM.jpg]

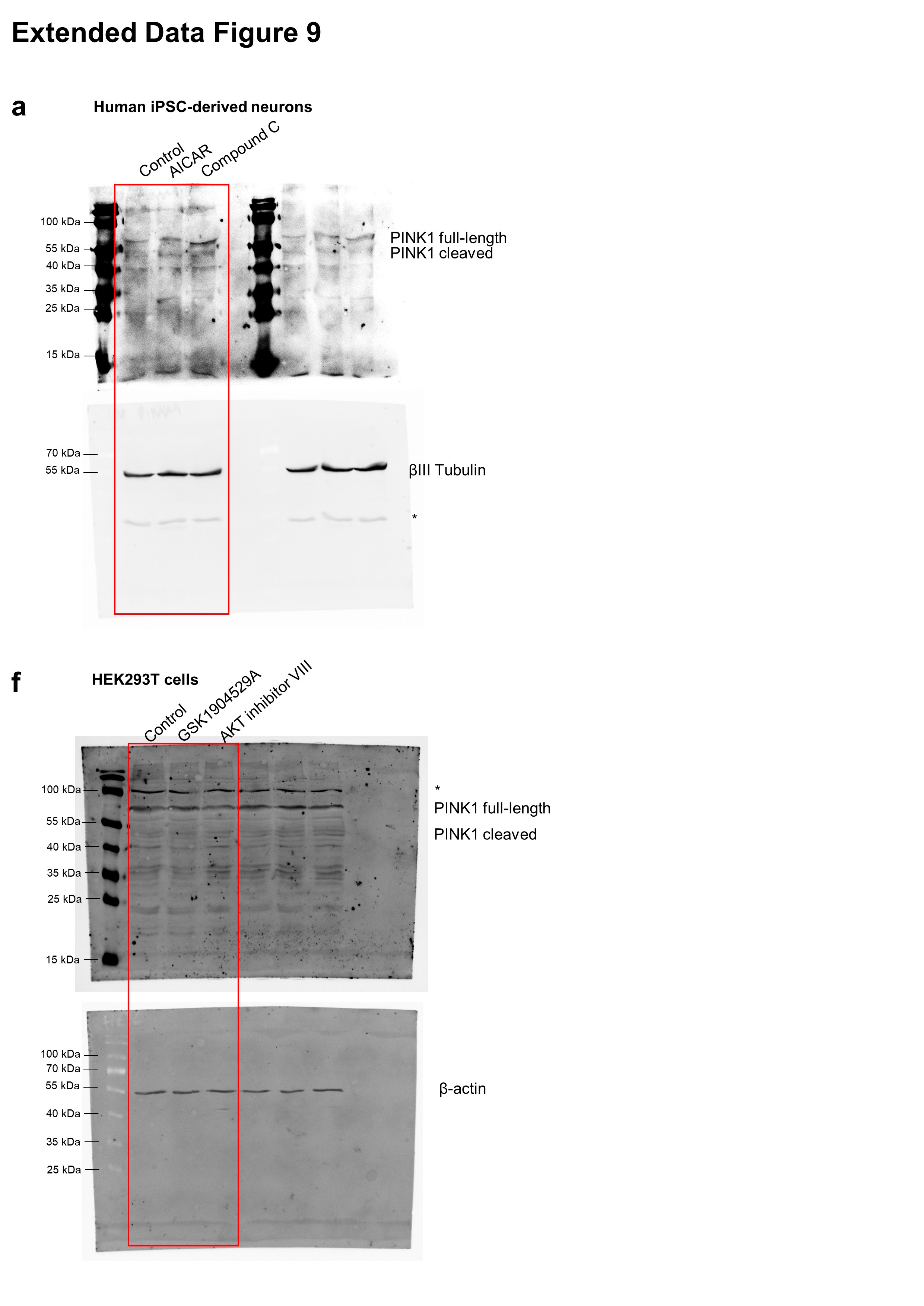

Supplement: Supplementary file 29 — Unprocessed western blots. [file 42255_2024_1007_MOESM29_ESM.jpg]
